# Stress-induced alteration of small extracellular vesicles drives amyloid-beta sequestration and exacerbates Alzheimer’s disease pathogenesis

**DOI:** 10.1186/s13195-026-02028-1

**Published:** 2026-04-11

**Authors:** Sadaqa Zehra, Sanskriti Rai, Komal Rani, Saumitra Dey Choudhury, Himanshu Rai, Suchismita Bhowmik, Nitin Mohan, Anu Gupta, Prasun Chatterjee, Thota Jagadeshwar Reddy, Neerja Rani, Gyan Prakash Modi, Fredrik Nikolajeff, Saroj Kumar

**Affiliations:** 1 Department of Biophysics, All India Institute of Medical Sciences, New Delhi, 110029 India; 2 Department of Pathology and Laboratory Medicine, All India Institute of Medical Sciences Bibinagar, Hyderabad, 508126 India; 3 Microscopy and Imaging sub-facility, Centralized Core Research Facility (CCRF), All India Institute of Medical Sciences, New Delhi, 110029 India; 4 Department of Pharmaceutical Engineering & Technology, Indian Institute of Technology BHU, Varanasi, 221005 India; 5 Biological Sciences & Bioengineering Department, Indian Institute of Technology-Kanpur, Kanpur, Uttar Pradesh 208016 India; 6 Department of Neurology, All India Institute of Medical Sciences, New Delhi, 110029 India; 7 Department of Geriatric Medicine, All India Institute of Medical Sciences, New Delhi, 110029 India; 8 Analytical Department, CSIR-Indian Institute of Chemical Technology, Hyderabad, 500007 India; 9 Department of Anatomy, All India Institute of Medical Sciences, New Delhi, 110029 India; 10 Department of Health, Education and Technology, Lulea University of Technology, Lulea, 97187 Sweden

**Keywords:** Alzheimer’s disease (AD), Amyloid-β aggregates, small Extracellular vesicles (sEVs), Oxidative stress

## Abstract

While small extracellular vesicles (sEVs) are implicated in amyloid-β trafficking, the mechanisms governing their interaction with Aβ aggregates and plaque formation remain unresolved. Here, we report a sEVs undergo dynamic structural remodelling in response to stress, enabling selective binding to Aβ aggregates- a phenomenon absent under normal physiological conditions. Using multimodal stressors, including mechanical (ultrasonication/agitation), physical (hyperthermia), and biological (oxidative damage), we demonstrate that stress-modified sEVs exhibit high-affinity binding to small Aβ aggregates (SA) through scaffold reorganization, as validated by Lipidomic analyses, single-molecule fluorescence microscopy, and quantitative colocalization assays. Crucially, these remodelled sEVs act as potent carriers, enhancing SA internalization by neuronal cells in vitro. Strikingly, in post-mortem Alzheimer’s disease (AD) brains and APP-PS1 transgenic mice, sEVs were spatially enriched at amyloid plaque margins, suggesting a direct role in Aβ sequestration and plaque expansion. Consistent with clinical relevance, sEVs isolated from AD patients exhibited an intrinsic SA-binding capacity, recapitulating stress-induced interactions observed experimentally. Our findings reveal that stress-primed sEVs function as pathological chaperones, binding to and internalizing Aβ aggregates, thereby accelerating plaque nucleation and disease progression. This study provides the first evidence of stress-mediated sEV plasticity as a critical driver of Aβ pathology, redefining therapeutic strategies targeting extracellular vesicle biology in neurodegenerative disorders.

**Supplementary Information:**

The online version contains supplementary material available at 10.1186/s13195-026-02028-1.

## Background

Alzheimer’s disease (AD) is the most prevalent form of dementia, associated with an aging population, and is a major worldwide health problem [1]. The underlying neuropathology of AD is strongly attributed to the accumulation of intracellular neurofibrillary tangles made of hyperphosphorylated tau protein and extracellular amyloid-beta (Aβ) plaques [2]. Aβ peptides, particularly Aβ42, have been reported to be prone to misfolding and aggregation [3].

Small extracellular vesicles (sEVs) are nano-sized, membrane-bound particles that are secreted by virtually all cell types into the extracellular space [4]. Typically ranging from 30 to 150 nanometers in diameter, sEVs have been extensively studied in recent years due to their essential role in intercellular communication [5]. The specific molecular content of sEVs comprising proteins, lipids, and various RNA species, including mRNAs and microRNAs, is dictated by the originating cell type and its physiological or pathological state and has profound relevance in different physiological processes and pathological conditions, including neurodegenerative diseases like AD [6]. The sEVs have been shown to carry amyloid-beta (Aβ) and tau proteins, key pathological markers of the disease, facilitating their spread across different brain regions and contributing to the progressive nature of neurodegeneration observed in Alzheimer’s disease [7, 8]. The sEVs also function as Aβ scavengers by ensnaring Aβ through the glycosphingolipids on their surface [9]. In-depth electron microscopy analyses of the brains of transgenic APP mice have revealed the presence of neuronal endosomes that contain Aβ, notably MVBs [10]. Recent studies have shown that extracellular plaques primarily arise from intraneuronal β-amyloid accumulation within membrane tubules, forming a central amyloid “core” which then degenerates to produce classical senile plaques [11]. In 2006, Rajendran et al. observed that APP-transfected cells released small amounts of Aβ in association with exosomes and they also reported the exosomal marker Alix to be around human amyloid plaques, suggesting that exosome-associated Aβ plays a role in plaque formation [12]. In the context of exosomes, it is presumed that Aβ is topologically associated with the surface membrane [13]. Immuno-electron microscopy analysis of exosomes isolated from N2a cells expressing APP revealed surface-bound Aβ [14]. Research into the molecular mechanism underlying the binding of Aβ to exosomes has been ongoing. Recent findings indicate that Aβ interacts with glycosphingolipids (GSLs), which tend to collect on the membrane’s outer layer, exposing their glycans to the outside environment. Aβ recognizes and binds to these GSL clusters [15]. There is heterogeneity in scientific studies discussing the association of EVs with amyloid-β, where some groups have reported the role of EVs in reducing the rate of Aβ [1–40] fibril formation by posing interference in the fibril elongation step [16]. In addition to transporting Aβ and tau, sEVs in AD are thought to participate in the neuro-inflammatory response by transporting pro-inflammatory molecules, sEVs activate microglia and astrocytes, exacerbating neuronal damage [17, 18].

Alzheimer’s disease progression is attributed to multiple pathophysiologic mechanisms [19]. Studies have discussed the role of oxidative stress on AD progression and numerous antioxidant drugs have been in trial for AD therapeutics [20–23]. Oxidative stress increases EV production by promoting MVB degradation [24] and alters EV cargo composition, affecting oxidative states in recipient cells [25]. It also contributes to β-sheet-rich fibril formation of Aβ42 [26]. Additionally, oxidative stress oxidizes amyloid-β into toxic oligomers and alters EV lipid bilayer conformation through lipid peroxidation, driving disease progression [27, 28]. We have studied the EV-Aβ association under mechanical, physical, and biological stress conditions, explaining how oxidative stress acts as a disease-relevant pathophysiological process that alters EV scaffold and enhances their affinity for Aβ.

## Results

### Characterization and validation of isolated EVs

**Fig. 1:**
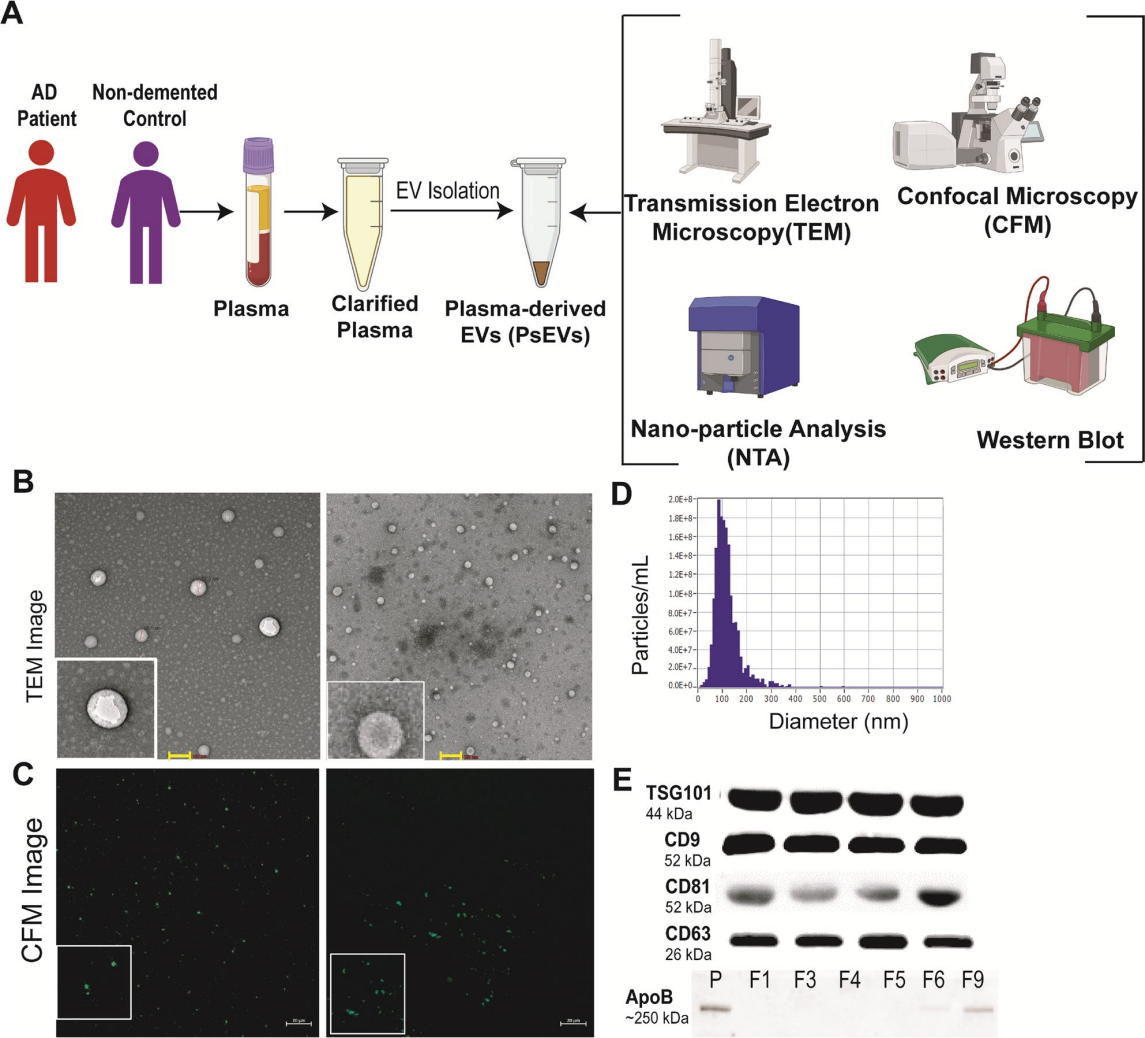
Graphical representation of workflow for isolation and characterization of Plasma-derived EVs (**A**). Characterization of isolated EVs through transmission electron microscopy (**B**), Scale bar= 100 nm; and confocal microscopy (**C**), Scale bar= 20 μm. Nanoparticle Analysis (NTA) showing the size distribution of EVs (nm) and particle concentration (particle/ml) (**D**); Western blot of EVs markers (CD9, CD63, CD81, Tsg101) and protein co-isolate (Apolipoprotein-B) where Plasma (P) and F [1–9] = SEC Fractions collected (**E**)

Plasma-derived sEVs (PsEVs) were successfully isolated, characterized, and validated (Fig. 1A). In Fig. 1, the plasma-derived sEVs were morphologically characterized by transmission electron microscopy (TEM) where spherical lipid bi-layered vesicles were observed in the size range of 65–80 nm in diameter (Fig. 1B, Supplementary Fig. 1A). PsEVs were also visualized by Confocal Microscopy using Alexa Fluor 488 tagged anti-CD9 antibodies (Fig. 1C, Supplementary Fig. 1B-C). The size distributions and concentration of plasma-derived sEVs were observed by nanoparticle tracking analysis. The mean concentration of plasma-derived sEVs was 5.0E + 10 particle/ml (Fig. 1D). Western blot validated the presence of sEV-specific markers (surface marker: CD9, CD63, CD81, and luminal marker: TSG101) in the isolated sEVs (Fig. 1E, Supplementary Fig. 1D). We also assessed the level of co-isolated contaminating protein like apolipoprotein in our sample (Size-exclusion chromatography fractions: F3-F6) (Fig. 1E). Finally, for the ease of readers, we will be using the acronym (EVs) while addressing Plasma-derived sEVs (PsEVs) hereon.

### Characterization of different Amyloid-β aggregates

**Fig. 2:**
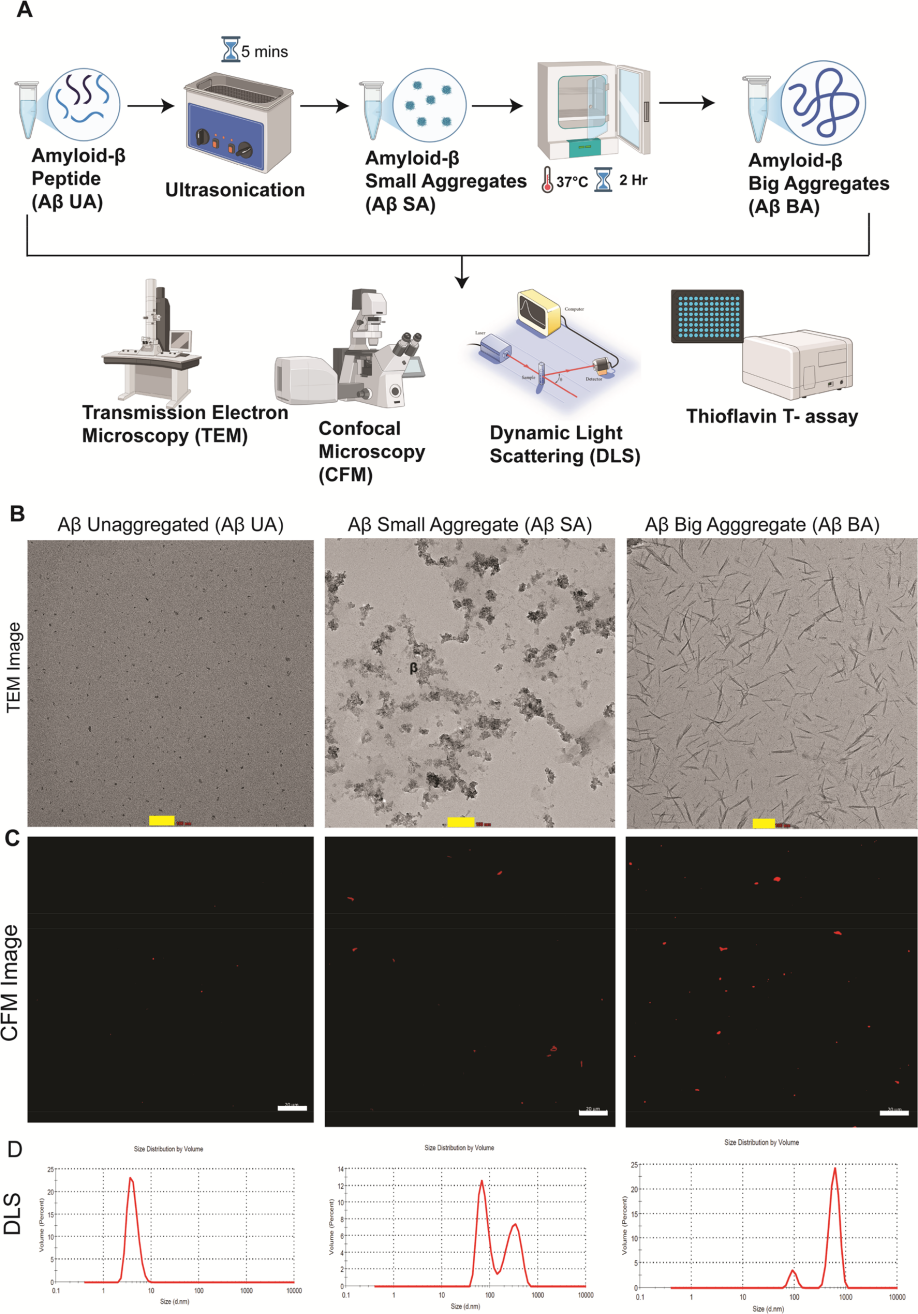
Graphical representation of workflow for different amyloid-β aggregate preparation and characterisation **A**. Characterization of different amyloid-β aggregate forms. Transmission electron micrographs (**B**), Scale bar= 100 nm; showed distinct morphology of the different Aβ aggregates. Confocal micrographs show increasing signal intensity according to Aβ aggregate size **C**. Scale bar= 20 μm. Dynamic light scattering analysis (**D**) A= Unaggregated Aβ form (~ 10 nm); *SA* Small Aβ aggregates (~ 100 nm); *BA * Big Aβ aggregates (~ 1000 nm)

To mimic different amyloid-β species relevant to AD pathology, we prepared different-sized aggregates of Aβ-42 and confirmed their structural morphology and size distribution, through transmission electron microscopy, confocal microscopy, dynamic light scattering analysis, and Thioflavin-T (ThT) fluorescence. Lyophilized Amyloid-β was resuspended in 60mM NaOH with pH adjusted to 7.4; this starting solution was termed unaggregated amyloid beta peptides (UA) and immediately stored at -80℃ until further use. Small amyloid beta aggregates (SA) were prepared by ultrasonication, and fibrillar aggregates or big aggregates (BA) were prepared by ultrasonication followed by mild agitation for two hours (Fig. 2A). For the ease of readers, we will be using the acronym (UA) for unaggregated amyloid beta peptides, (SA) for small amyloid beta aggregates (SA) were prepared by ultrasonication and (BA) while addressing fibrillar aggregates or big aggregates (BA) were prepared by ultrasonication followed by mild agitation for two hours hereon. TEM micrograph revealed the representative aggregated structures both in SA and BA samples. UA group did not form any large visible aggregates, while the SA group formed visible globular aggregates, and the BA group exhibited typical features of short-rod-like protofibrils (Fig. 2A, Supplementary Fig. 2B). Following this, we also incubated all three groups, i.e., UA, SA, and BA, with AlexaFluor647 Anti-Aβ42 Antibodies and observed under 40X magnification in Confocal Microscope (CFM) (Fig. 2C). The CFM images reveal the distinct variations in fluorescent signals between these groups (Supplementary Fig. 2B-C). Finally, we also assessed the size distribution using DLS, which further supported our claims on the nomenclature of the prepared aggregates based on their size (Fig. 2D). DLS findings revealed the median size for UA, SA and BA was approximately 9, 100 and 1000 nm respectively (Fig. 2C). Finally, we assessed the ThT fluorescence for each BA, SA and UA groups and observed enhanced fluorescence in BA group as a result of ThT binding to the β-sheet in the fibrillar structure of BA (Supplementary Fig. 2D).

### Oxidative stress alters sEVs’ driving amyloid-β-EVs association

**Fig. 3:**
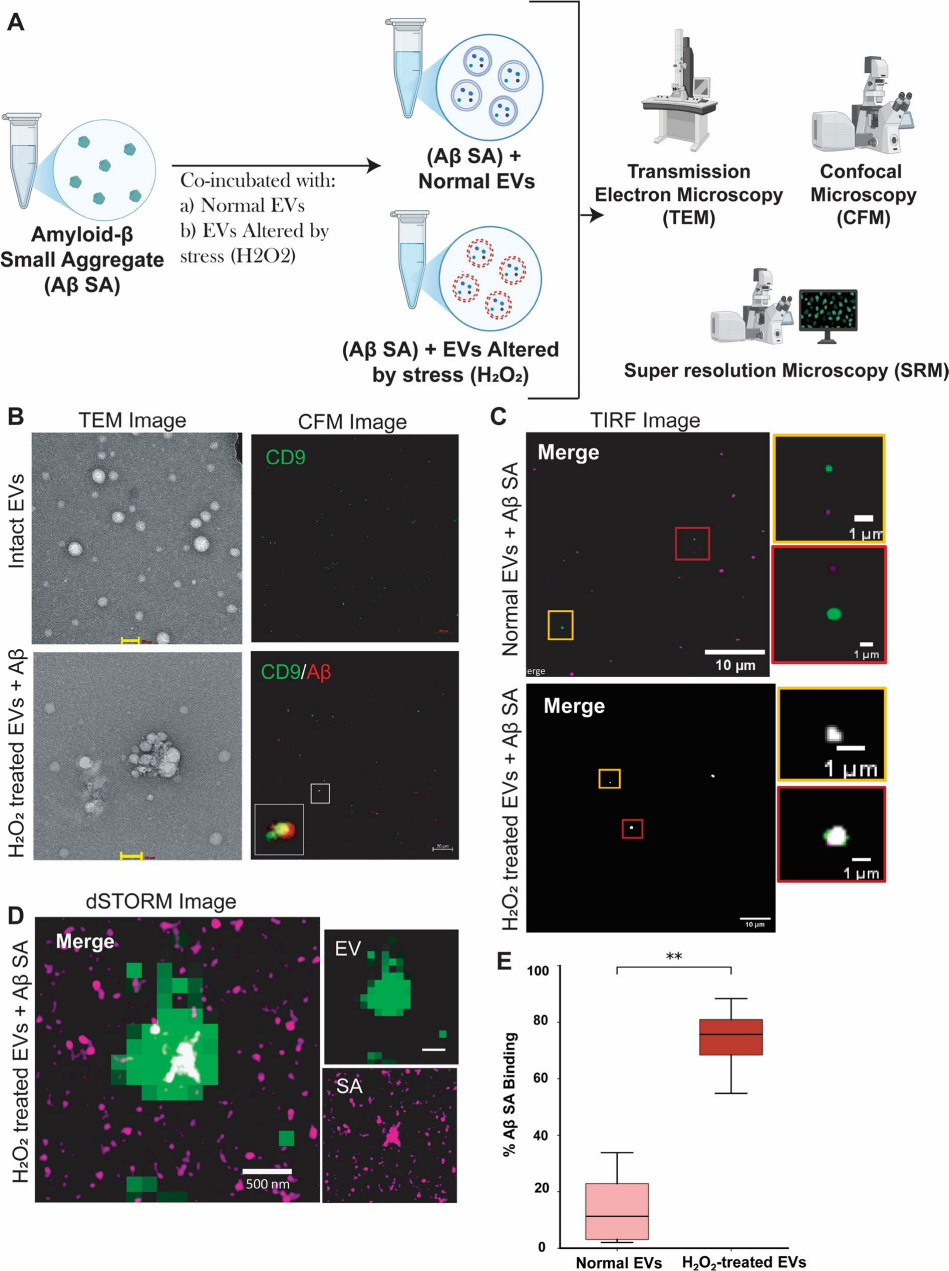
Graphical representation of workflow **A**. TEM and CFM micrograph shows aggregated structure in H2O2-treated EVs and Aβ aggregates (**B**), TEM Scale bar= 100 nm. CFM Scale bar= 20 μm. Comparison of interaction between normal and H_2_O_2_-treated EVs by TIRF micrographs **C**. Colour white is the merged signal of EVs (Green) and Aβ (red). dSTORM Image shows EV-Aβ coaggregates **D**. Colour white is the merged signal of EVs (Green) and Aβ (Magenta). Percentage of AβSA bound to EVs (**E**), calculated from the ratio of white EV-AβSA colocalized area to total AβSA area, presented as mean ± SD (*n* = 4). H₂O₂-treated EVs showed significantly higher AβSA binding compared to Normal EVs (unpaired t-test, *p* < 0.005)

Oxidative stress plays a well-established role in the pathogenesis of Alzheimer’s disease, as the neurons undergoess oxidative damage resulting from excessive production of reactive oxygen species (ROS), which triggers oxidative stress and inflammatory responses, thereby accelerating AD progression. Hydrogen peroxide (H_2_O_2_) is considered a major ROS contributor and is widely used in vitro model for oxidative stress in the context of several progressive neurodegenerative diseases. Oxidative stress conditions also induce substantial modifications in EV membrane composition, leading to altered biophysical properties that facilitate the sequestration and aggregation of pathogenic proteins such as amyloid-β [29]. Therefore, in our study, H_2_O_2_-induced stress was adopted as an oxidative stress model. EVs that were not exposed to any stress viz.; (H_2_O_2_ and Ultrasonication) served as the control group (Fig. 3A). We subjected sEVs to varying concentrations of H₂O₂ (100 µM, 10 µM, and 1 µM) for 24 h, where we observed that higher concentrations led to the formation of large aggregated masses (Supplementary Fig. 3A). Therefore, we selected the minimal dose of 1 µM for inducing oxidative stress. After incubating EVs with a 1µM H_2_O_2_ solution for 24 h, we compared the H_2_O_2_-treated EVs with ultrasonicated sEVs (Supplementary Fig. 3B). TEM images revealed the structural morphology of EVs was distorted in H₂O₂-treated group compared to that of control EVs indicating alteration in sEVs structural integrity. Similarly, Confocal Microscope (CFM) Fig. 3B, and Total Internal Reflection (TIRF) images (Fig. 3C) also supported TEM findings whereby normal EVs do not form coaggregates with Aβ, while H_2_O_2_ -treated EVs associate with Aβ. These altered EVs showed self-aggregation, even at low temperatures (4℃) (Supplementary Fig. 3C). We also observed this EV-Aβ coaggregates by the direct stochastic optical reconstruction microscopy (dSTORM) (Fig. 3D), whereby the percentage of AβSA bound to EVs was significantly (*p* < 0.005) higher in H_2_O_2_-treated group compared to the control (Untreated) Group (Fig. 3E).

**Fig. 4:**
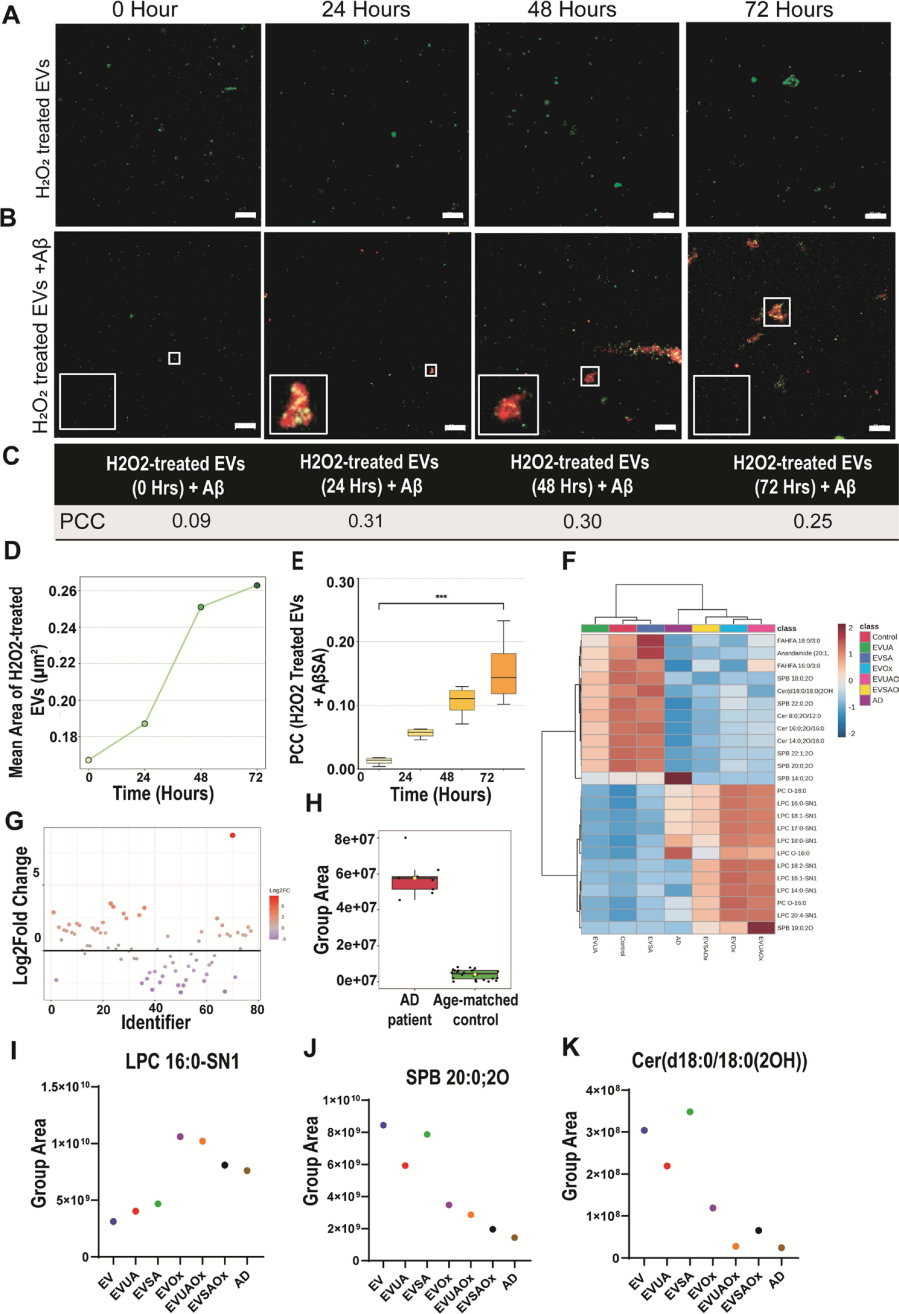
Temporal assessment of small amyloid-β aggregates and EV association at low temperature 4℃. An increase in H2O2-treated EV size was observed **A**. Increased colocalization in EV-Aβ signals suggests an active sequestering by altered EVs in a time-dependent manner (**B**) and Colocalization coefficient **C**. Colour yellow is the merged signal of EVs (Green) and Aβ (red). Scale bar= 20 μm. **D** Mean Aggregate size (µm²) at each timepoint (0 h: 0.168 μm²; 24 h: 0.188 μm²; 48 h: 0.250 μm²; 72 h: 0.263 μm²). PCC of H₂O₂-treated EVs (**E**) incubated with AβSA over time. PCC values (mean ± SD, *n* = 4) were calculated using Costes-thresholded colocalization (FIJI/JACoP).One-way ANOVA: *p* < 0.001. Lipidomic analysis of EV lipids in different groups **F**. Volcano plot of DE Lipids (**G**), Group area of oxidized glycerophospholipid between Control and AD patient **H**. Top dysregulated lipid: LPC (**I**), SPB (**J**) and Ceramide (**K**)

Furthermore, to monitor the effect of oxidative stress on EV-Aβ interaction, we subjected EVs to 1µM H_2_O_2_ stress for 24, 48 and 72 h and incubated with Aβ SA for 2 h at 4℃ (Fig. 4A). CFM images revealed an increase in EV signal intensity, indicating self-aggregation due to structural alterations in EVs. We observed a gradual increase in colocalization of EV-Aβ signals, indicative of active sequestration by the altered EVs over time (Fig. 4B). An increase in EV size further supported these observations. (Supplementary Fig. 3D). Pearson’s Correlation Coefficient was used to estimate the measure of colocalization for different time points which were; 0 h = 0.09, 24 h = 0.31, 48 h = 0.30 and 72 h = 0.25) (Fig. 4C, Supplementary Fig. 3E). Finally, an increase in mean aggregate size from 24 h to 72 h (Fig. 4D) together with a significant increase in PCC values from 24 to 72 h (*p* < 0.001) suggests increased association between altered EVs and Aβ SA (Fig. 4E).

A comparative analysis of the lipidome in Alzheimer’s disease (AD)-derived extracellular vesicles (EVs), normal EVs, and hypoxia-treated EVs with SA was conducted using untargeted lipidomic analysis (Fig. 3.2F). Notably, lipids greatly differed between Control (Untreated) EVs and EVs exposed to oxidative stress (Fig. 3.2G). Lipidomic analysis of AD EVs revealed elevated levels of Oxidized Glycerophospholipid (Fig. 3.2H), which forms the structural framework of EV membranes [30]. This analysis also revealed that lipid classes such as Lysophosphatidylcholine (LPC) (Fig. 3.2I), Sphinganine (SPB) (Fig. 3.2J), and Ceramide (Cer) (Fig. 3.2K) were highly dysregulated in stress-altered EVs compared to normal EVs and showed similar dysregulation patterns to those seen in AD-derived EVs. These results highlight the effects of oxidative stress on lipids present in the EV membrane, which further influence Aβ sequestration and aggregation.

### Amyloid-β aggregates size regulate their association with EVs

**Fig. 5:**
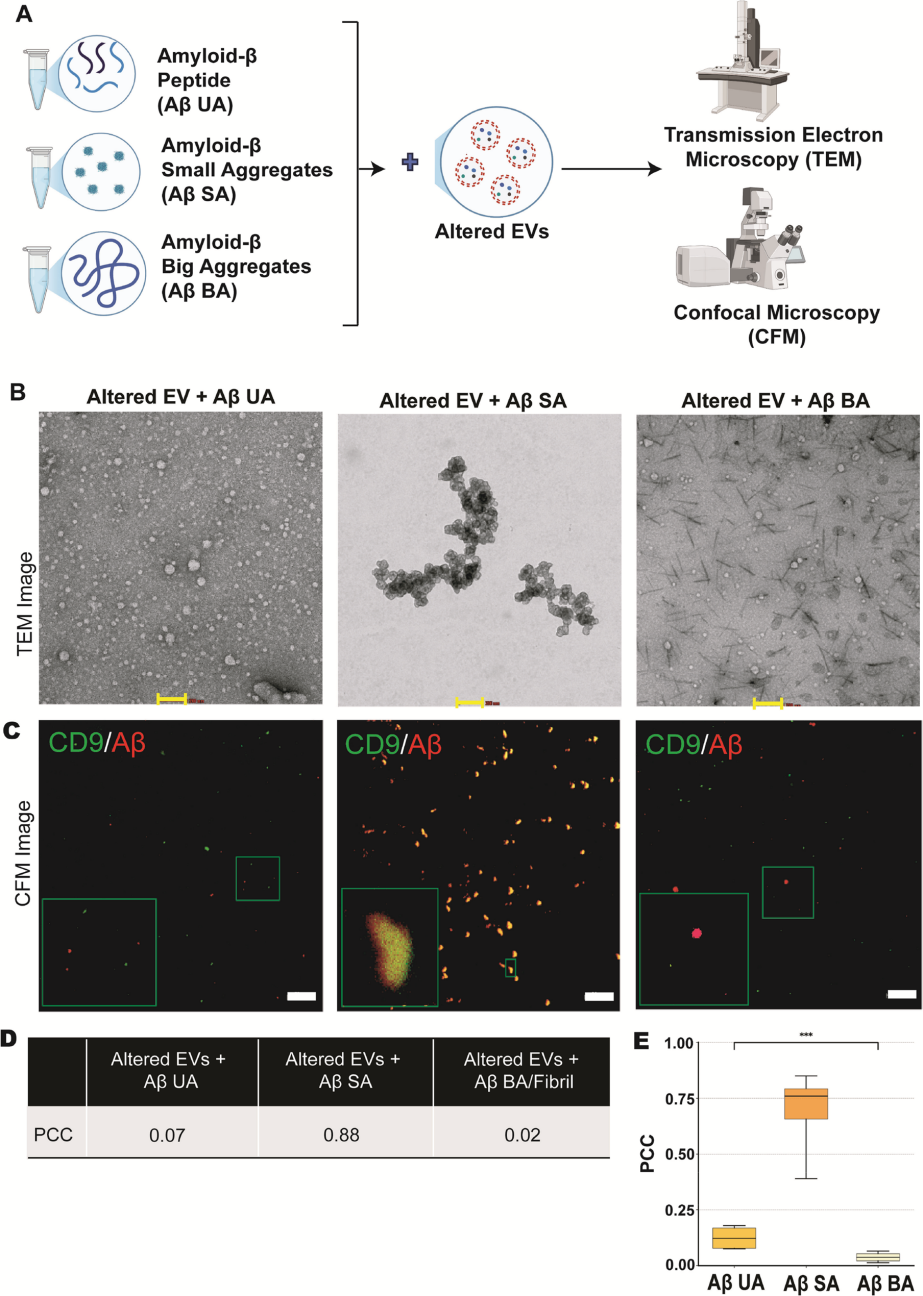
Graphical representation of workflow **A**. In-vitro assessment of different forms of amyloid-β and stress-altered EV association. (Aβ42 UA= Unaggregated, Aβ42 SA= Small Aggregates, Aβ42 BA/Fibril = Big aggregates). TEM micrograph shows the distinct aggregated structures (**B**), Scale bar= 100 nm. Confocal microscopy shows enhanced SA binding to EVs (**C**), Scale bar= 20 μm. Colour yellow is the merged signal of EVs (Green) and Aβ (red). PCC Values for all 3 groups **D**. PCC analysis of EV colocalization with AβUA, AβSA, and AβBA. PCC values (mean ± SD, *n* = 4) were calculated using Costes-thresholded colocalization (FIJI/JACoP). EV + AβUA: 0.125 ± 0.051; EV + AβSA: 0.690 ± 0.200; EV + AβBA: 0.038 ± 0.023. **E** One-way ANOVA: *p* < 0.001

After examining the association between EVs and Amyloid-β species with exposure to oxidative stresses, we further assessed whether this association depends on the size of Aβ itself. We prepared different-sized aggregates of Aβ-42, specifically assigned them as UA, SA, and BA following extensive characterization by TEM, CFM, DLS, and ThT assay (Fig. 2). Next, we incubated each group viz.; UA, SA, and BA with stress-altered EVs to evaluate the association (Fig. 5A). We examined whether the different-sized aggregates affected their association with EVs using TEM and CFM. The TEM images revealed a characteristic cluster in the EVs and SA group, which was not present in either the UA or BA group, suggesting EVs had a preferential association with the SA group (Fig. 5B, Supplementary Fig. 4A). TEM images of EVs with UA and BA showed no association between EVs and Aβ. Consequently, confocal microscopy confirmed that the SA has enhanced binding toward EVs (Fig. 5C), as depicted by the colocalized signal (Yellow colour) from both EVs and Aβ-42 antibodies signals. CFM images did not reveal any colocalized signals for EVs with UA and BA groups (Supplementary Fig. 4B). Co-localization analysis was used to estimate the measure of colocalization Pearson’s Correlation Coefficient for the EV + SA group was 0.88 suggesting more association between EVs and SA compared to UA (PCC = 0.07) and BA group (PCC = 0.02) (Fig. 5D, Supplementary Fig. 4C). Furthermore, PCC analysis of this colocalization was significantly higher in Aβ-SA group compared to others (*p* < 0.001) (Fig. 5E). This supports the conclusion that SA preferentially binds to EVs compared to other amyloid-β aggregates.

### Assessment of EVs-Aβ association in a time-dependent manner

**Fig. 6:**
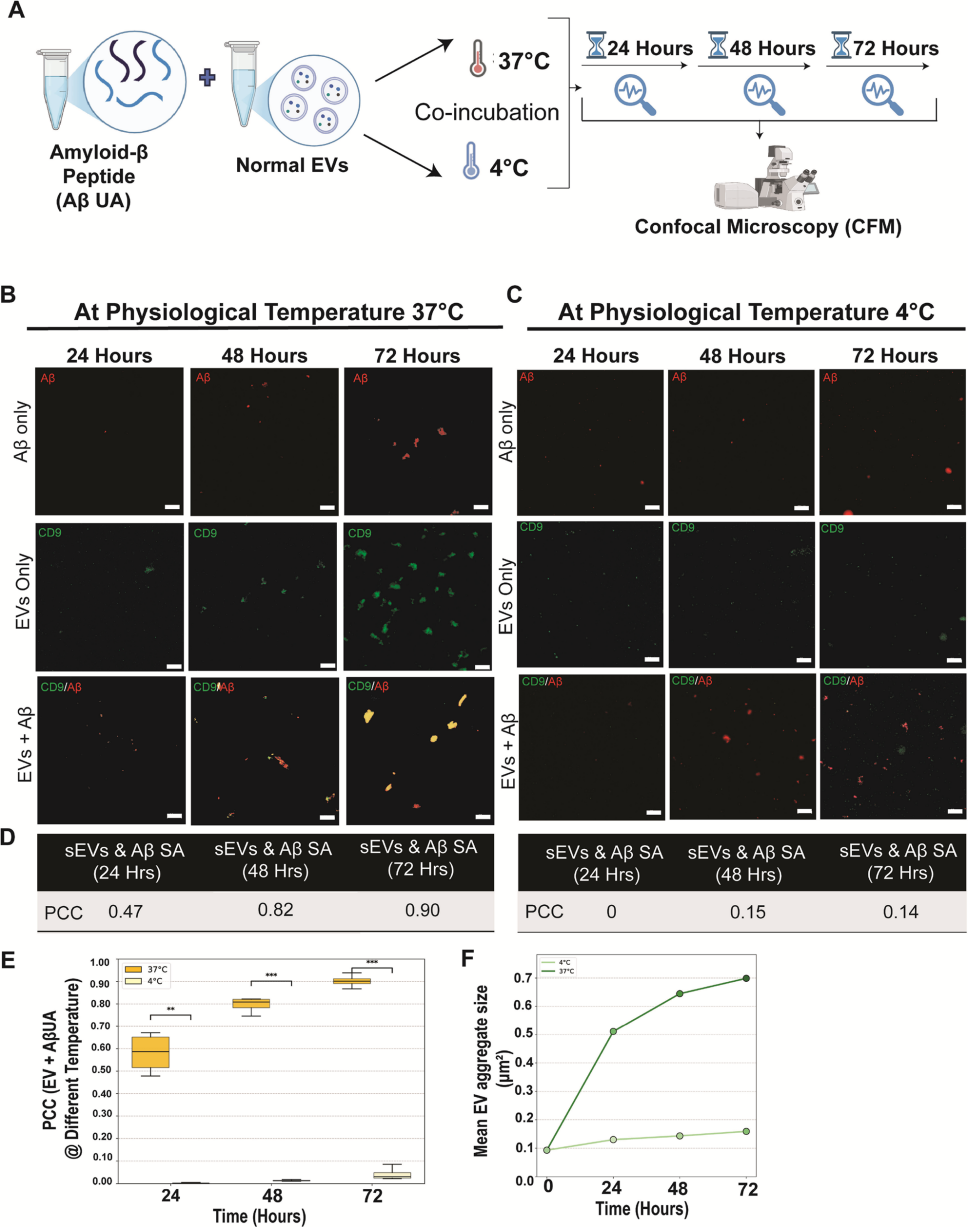
Graphical representation of workflow **A**. Temporal assessment of different forms of amyloid-β (Unaggregated) and EV association: at physiological temperature 37℃ (**B**)- Colour (yellow) represents the merged image of EVs (Green) and Aβ (Red) signal, and low temperature 4℃ **C**. Colour yellow is the merged signal of EVs (Green) and Aβ (red). Scale bar= 20 μm. D) PCC at 37 °C & 4 °C, E) PCC increased over time (24 h: 0.580 ± 0.089; 48 h: 0.796 ± 0.034; 72 h: 0.902 ± 0.030), whereas values at 4 °C remained low (24 h: 0.0025 ± 0.0017; 48 h: 0.0138 ± 0.0030; 72 h: 0.0423 ± 0.0294). Aggregate size (µm²) (**F**) was quantified values represent mean aggregate size at each timepoint (0, 24, 48, 72 h) for EVs incubated at 37 °C (0 h: 0.093; 24 h: 0.511; 48 h: 0.645; 72 h: 0.699 μm²) and 4 °C (0 h: 0.093; 24 h: 0.130; 48 h: 0.143; 72 h: 0.159 μm²)

There is an existing ambiguity regarding the effect of EVs on extracellular amyloid-β. In line with our observations from significant findings pertaining to EVs-Aβ association as assessed by TEM and CFM, we proceeded with studying the EVs and amyloid-β incubated together at different time intervals viz., 24, 48, and 72 h at physiological temperature of 37℃ and low temperature 4℃ as control (Fig. 6A). We observed a gradual increase in the size of amyloid-β aggregates at 37℃, indicating active oligomerization over time (Fig. 6B). Similarly, we also noted an increase in EV signal intensities, possibly related to EV self-aggregation at 37 ℃ (Supplementary Fig. 5A). Additionally, we also repeated the same experiment at 4℃ (Fig. 6C), where low signal intensity was observed in both EVs and amyloid-β. It is well known that the storage conditions significantly influence the integrity and functionality of extracellular vesicles (EVs) [31, 32]. Compared to PCC values at 37℃: 0.47 at 24 h, 0.82 at 48 h, and 0.90 at 72 h, at 4℃, no close association was observed when EVs and Aβ were incubated together, as indicated by PCC values of 0.0 at 24 h, 0.15 at 48 h, and 0.14 at 72 h (Fig. 6D). These higher signal intensities at 37 °C could be attributed to amyloid-β sequestration mediated by EV-fragmentation (Supplementary Fig. 5B). Finally, the EV-Aβ association also increased over time, as shown by the PCC values at 37℃ compared to values at 4℃ which remained low (Fig. 6E). Also, the mean aggregate size increased from 24 to 72 h (Fig. 6F).

These findings imply that the observed association may reflect active Aβ oligomerization and EV membrane conformational changes under higher temperature conditions as primary mediators behind the close association between EVs and Aβ. Additionally, EV aggregation appeared to contribute to Aβ sequestration, particularly in the 37 °C experimental group. Finally, we concluded that EVs membrane integrity is affected by temperature and influences its association with amyloid-β aggregates. EVs aggregation may promote amyloid-β aggregation, potentially making it a pathophysiological factor relevant to AD pathology.

### In vitro assessment of EVs-Aβ association in response to other stress conditions and with proteins implicated in other neurodegenerative disorders

**Fig. 7:**
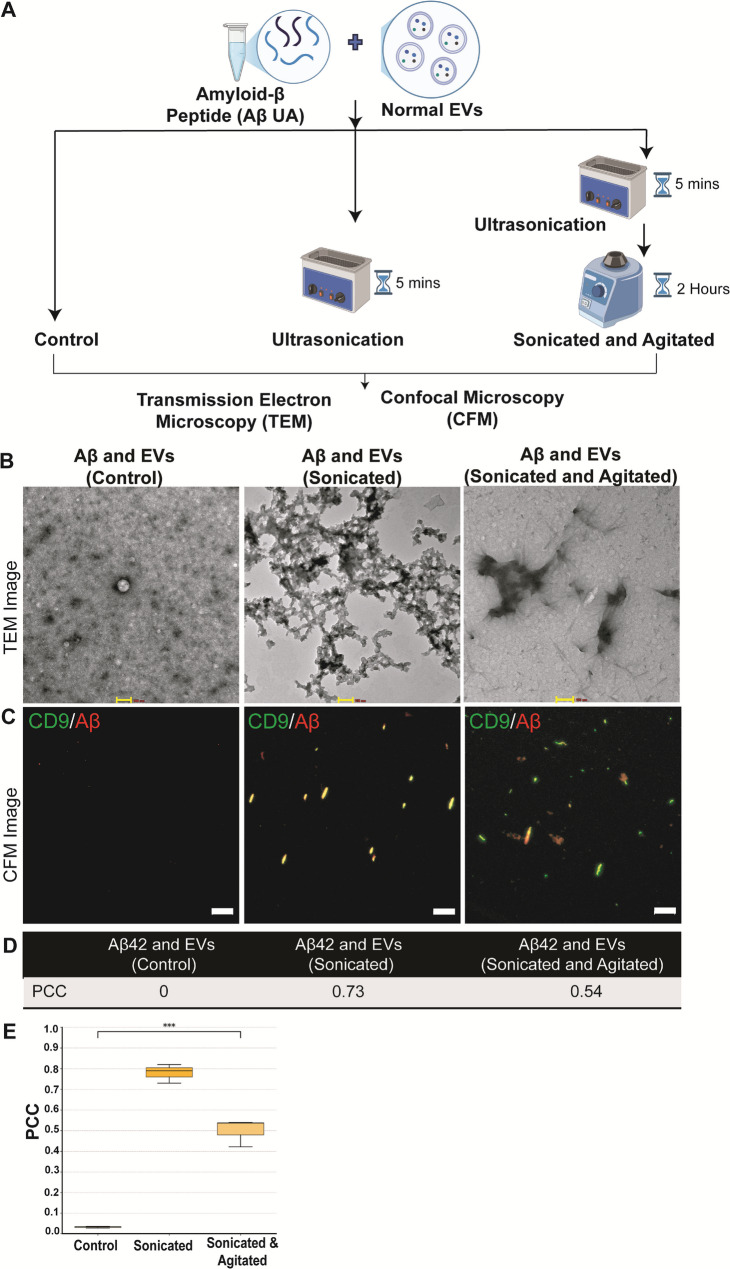
Graphical representation of workflow **A**. In-vitro assessment of amyloid-β (Unaggregated) and EV association. Imaging was performed at three key stages: Control (Without any stresses), Sonication only, and Sonication and agitation combined. TEM micrograph shows morphological characterization of different Aβ aggregated structures formed in the presence of EVs (**B**), Scale bar= 100 nm; CFM images for respective groups (**C**)and Colocalization analysis (**D**) showed maximum PCC in the post-sonication group (PCC = 0.73). Colour yellow is the merged signal of EVs (Green) and Aβ (red). Scale bar= 20 μm. PCC between CD9-Alexa 488 EVs and AβSA-Alexa 647. Control: 0.033 ± 0.005; Sonicated: 0.780 ± 0.046; Sonicated & Agitated: 0.499 ± 0.066 (*n* = 3). One-way ANOVA: *p* < 0.0001 (E)

EVs are known to carry amyloid-β as cargo and are often called in to cause the seeding effect in the progression of the AD pathology [15, 33]. Many studies have reported the amyloid beta aggregates to be topologically bound to the EVs surface membranes via glycolipid anchor. Transmission electron microscope (TEM) images have revealed that amyloid-β is attached to the surface of the EVs [13, 34]. To determine whether Aβ is merely anchored via a GPI anchor or engages in a more complex interaction with extracellular vesicles, we subjected both EVs and amyloid-β peptides to mechanical stresses such as ultrasonication and mild agitation. We incubated EVs and amyloid-β together and assessed their association by TEM. In addition, confocal microscopy was used for further morphological characterization using Antibody-specific, precise, and concise detection (Fig. 7A). EVs and amyloid-β did not show previously reported association when incubated without external mechanical stresses. However, when EVs and amyloid-β were ultrasonicated together, TEM images showed that amyloid-β was closely associated with EVs (Fig. 7B, Supplementary Fig. 6A), and confocal images also showed a significant overlap between the two signals (Fig. 7C). This could imply that the ultrasonication altered the EV membrane affinity to Aβ and also caused Amyloid-β to aggregate (Supplementary Fig. 6B). Furthermore, when we incubated EVs with amyloid-beta and then applied ultrasonication and mild agitation, we observed a distinct fibrillar structure similar to the BA structure in Fig. 2. However, this structure was not closely associated with the EVs. We also evaluated the fluorescence colocalization of two signals: AlexaFluor488 anti-CD9 and AlexaFluor647 anti-Amyloid-β. We measured Pearson’s correlation coefficient (PCC), which quantifies the overall association of two probes in an image [35]. We observed a PCC value of 0.73 in Sonicated samples and a PCC of 0.54 in sonicated and agitated samples (Fig. 7D). PCC values was significantly higher in groups subjected to mechanical stress (*p* < 0.0001) Fig. 7E. These findings demonstrate an association between EVs and amyloid-β. However, whether the structural conformation of EVs, amyloid-β, or a combination of both drives this association remained uncertain. Similarly, we conducted the experiment using alpha-synuclein, a hallmark protein associated with Parkinson’s disease. However, we did not observe any significant association with extracellular vesicles (EVs). (Supplementary Fig. 6C) This suggests that the observed selective interaction of altered EVs with amyloid-beta aggregates may be influenced by structural and size differences between the proteins.

### Altered EV-Aβ coaggregates are internalized by cells and also localized at Amyloid Plaques

**Fig. 8:**
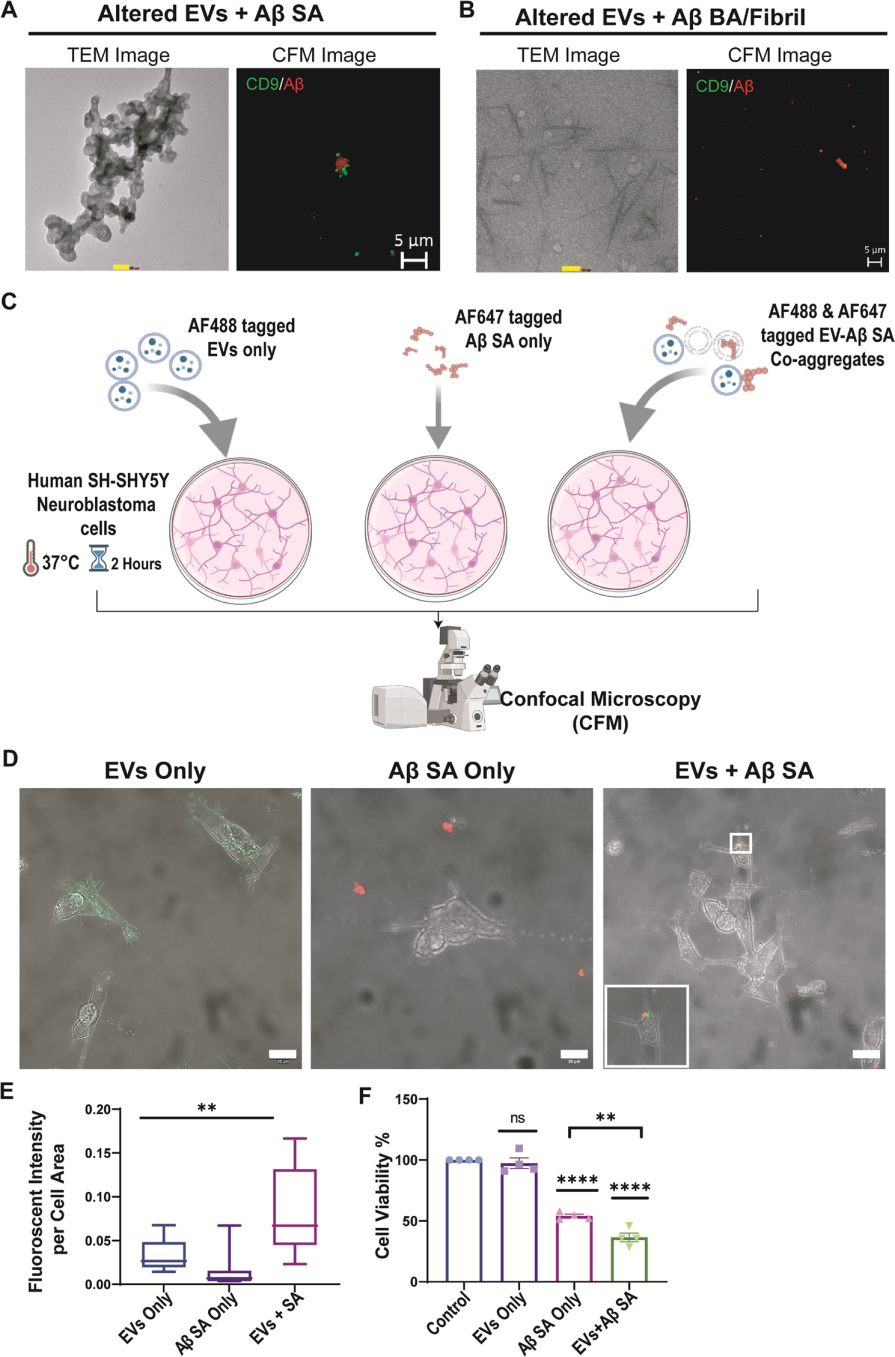
Comparison of interaction between EV and small amyloid-β aggregates (**A**) and big amyloid-β aggregates/Fibrils (**B**) at low temperature 4℃. The TEM, Scale bar= 100 nm;, and CFM micrograph shows the EVs sequestering the SA group as opposed to no association between BA/Fibril **B**. Graphical representation of workflow **C**. CFM image showing Alexa-Fluor-488 CD9 (Green) and Alexa-Fluor-647Amyloid-β (Red) signals of: EVs only; Aβ; and EVs and Aβ together **D**. Scale bar= 20 μm. Fluorescence intensity per cell was measured as Mean Fluorescence intensity/Area of ROI. Each condition included 3 cells per dish with a total of *n* = 9 cells per group. Statistical analysis using one-way ANOVA (Kruskal–Wallis test) indicated a significant difference (*p* < 0.001) **E**. Cell viability (**F**) 10,000cells/well were seeded and included 4 wells per plate with a total of *n* = 4. Statistical analysis using one-way ANOVA and multiple comparisons showing a highly significant effect (*p* < 0.00001)

Confocal images revealed that EVs sequester the amyloidβ aggregates, particularly the SA species (Fig. 8A). Consequently, co-incubation of BA with EVs did not reveal close proximation (Fig. 8B, Supplementary Fig. 7A). Similarly, the TEM micrograph shows the localization of amyloid-β aggregates (SA) around the EVs’ corona and when EVs +Aβ BA group, TEM images revealed the fibrils formed had clear filament twists (Fig. 8B). Notably, we concluded that the size of Aβ also influenced this association, where more pronounced aggregates were formed with SA compared to BA (Supplementary Fig. 7B).To assess whether or not the altered EVs and Aβ aggregates are uptake by cells, EVs alone, amyloid-β aggregates alone, and altered EVs and amyloid-β coaggregates were labeled using Alexafluor488 anti-CD9 and Alexafluor647 anti-amyloid-β antibodies and were added to SH-SY5Y cells culture media. The cells were then extensively washed and examined by confocal scanning microscopy (Fig. 8C). Similar to EVs, altered EVs and amyloid-β coaggregates were found to be internalized by the cells. However, Aβ aggregates alone did not show internalization (Fig. 8D, Supplementary Fig. 7C). Furthermore, the imaging of different optical sections of the cells revealed the presence of coaggregates in the plasma-membrane vicinity following their internalization (Supplementary Video 1). The internalization of only co-aggregates inside the cells could be attributed to EVs-mediated cellular uptake. Small red amyloid-β signals were detectable in confocal microscopy images outside the cells in amyloid-β alone, indicating that amyloid-β by itself does not enter cells. This suggests that only with the assistance of EVs can amyloid-β cross the cell membrane and reach the cell interior (Fig. 8D). Furthermore, fluorescence intensity per area was measured which showed a significant difference (*p* < 0.001) between the groups where EVs+Aβ SA group had the highest value (Fig. 8E). Cell viability was also measured, and %Cell viability was the lowest in EVs+Aβ SA compared to Aβ SA only (*p* = 0.0037), while both groups showed decreased cell viability compared to the untreated control (*p* < 0.00001). There was no significant difference between EVs only group and the untreated control (Fig. 8F).

**Fig. 9:**
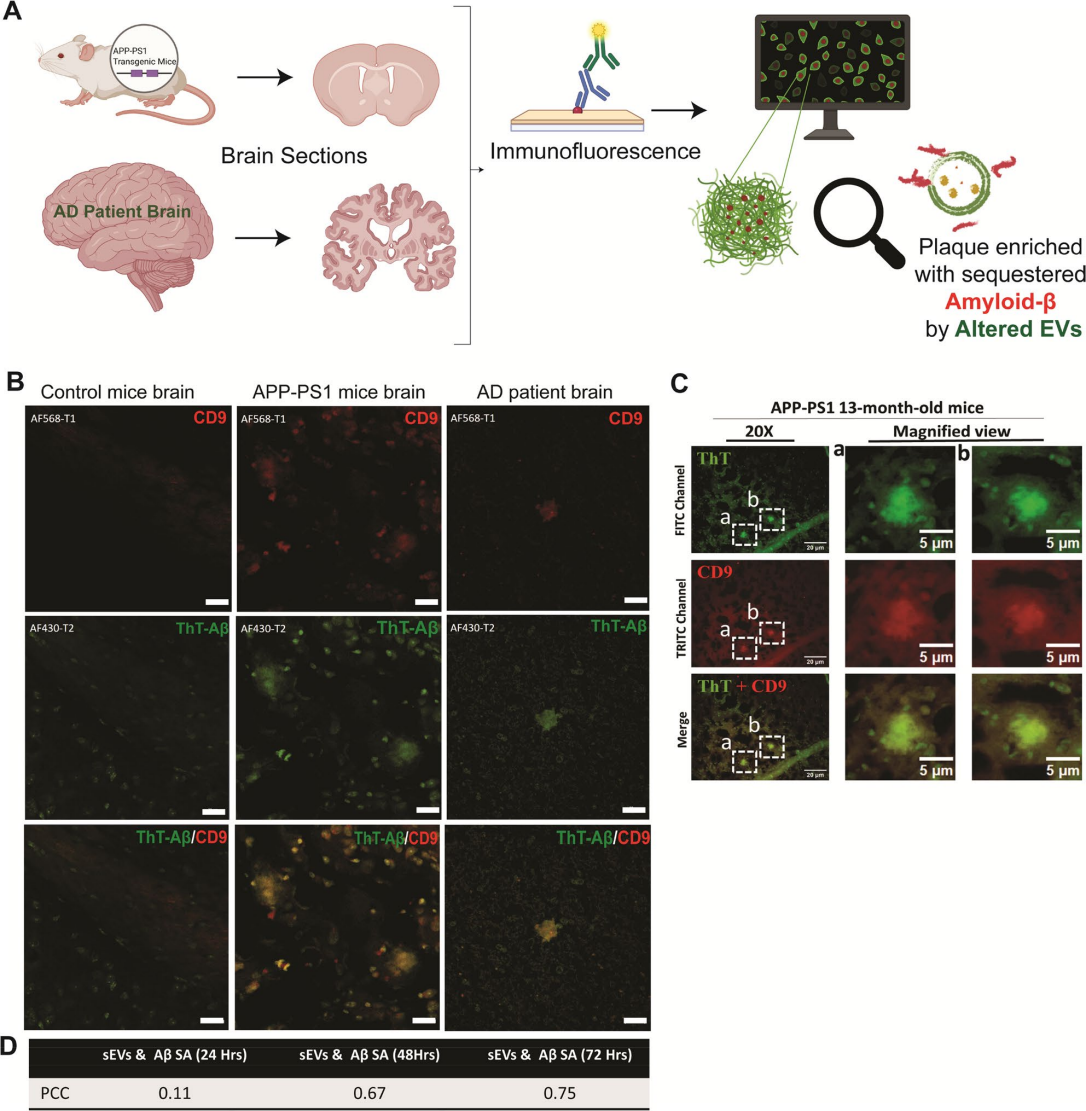
Graphical representation of workflow **A**. Immunohistological staining images of APP mouse brain sections with CD9 (EVs) and ThT (plaques) showing typical Amyloid- plaques with discrete CD9 signals (**B**) and respective colocalization coefficient **D**. In-vitro dual staining experimental images: (**C**) APP mice brain sections immunohistological staining with CD9 (antibody) and ThT stained Aβ. (*n* = 3 mice brains) (Scale bar 20 μm; Zeiss Confocal microscope and NIS-Elements BR 4.30.00.64-bit fluorescence microscope). Images were captured at (For ThT; λex = 450 nm and λem = 490 nm. CD9-Rodamine TRITC-conjugated; λex = 550 nm and λem = 570 nm). Scale bar= 20 μm

To determine whether these findings are relevant to AD pathology, we conducted immunohistochemistry using antibodies against the exosomal protein CD9 and performed ThT staining to label amyloid-β plaques in autopsy brain sections from Alzheimer’s disease patients, APP mice, and control mice (Fig. 9A). The CD9 EV marker signal was observed surrounding small Aβ plaques, while the ThT signal was dispersed within the plaques in brain sections from both AD patients and APP-PS1 mice. In contrast, ThT staining was largely absent in the brain sections of control subjects (Fig. 9B, ). Similarly, fluorescent images of the APP-PS1 mice brain reveal significant colocalization and accumulation of CD9 proteins in and around amyloid-β plaques (Fig. 9C). Pearson’s Correlation Coefficient for CD9-Aβ fluorescent labeled brain sections for: control mice were 0.11, APP-PS1 mice brain = 0.67, and for human AD brain = 0.75, suggesting enrichment of EVs around Amyloid-β plaques (Fig. 9D, Supplementary Fig. 7D). Extracellular deposition of Amyloid-β leads to the formation of senile plaques and characteristic exosomal CD-9 labeling observed throughout the vicinity of plaques provides a plausible mechanistic explanation that the altered EVs sequester small amyloid-β.

Congruent to the earlier findings reporting the enrichment of exosomal proteins around amyloid plaques in AD patients, where it is involved in plaque formation, we further add that the altered sEVs preferentially sequester small amyloid-β aggregates and aid in plaque formation.

### Assessment of sEVs-Aβ co-aggregates in circulating plasma-derived sEVs

**Fig. 10:**
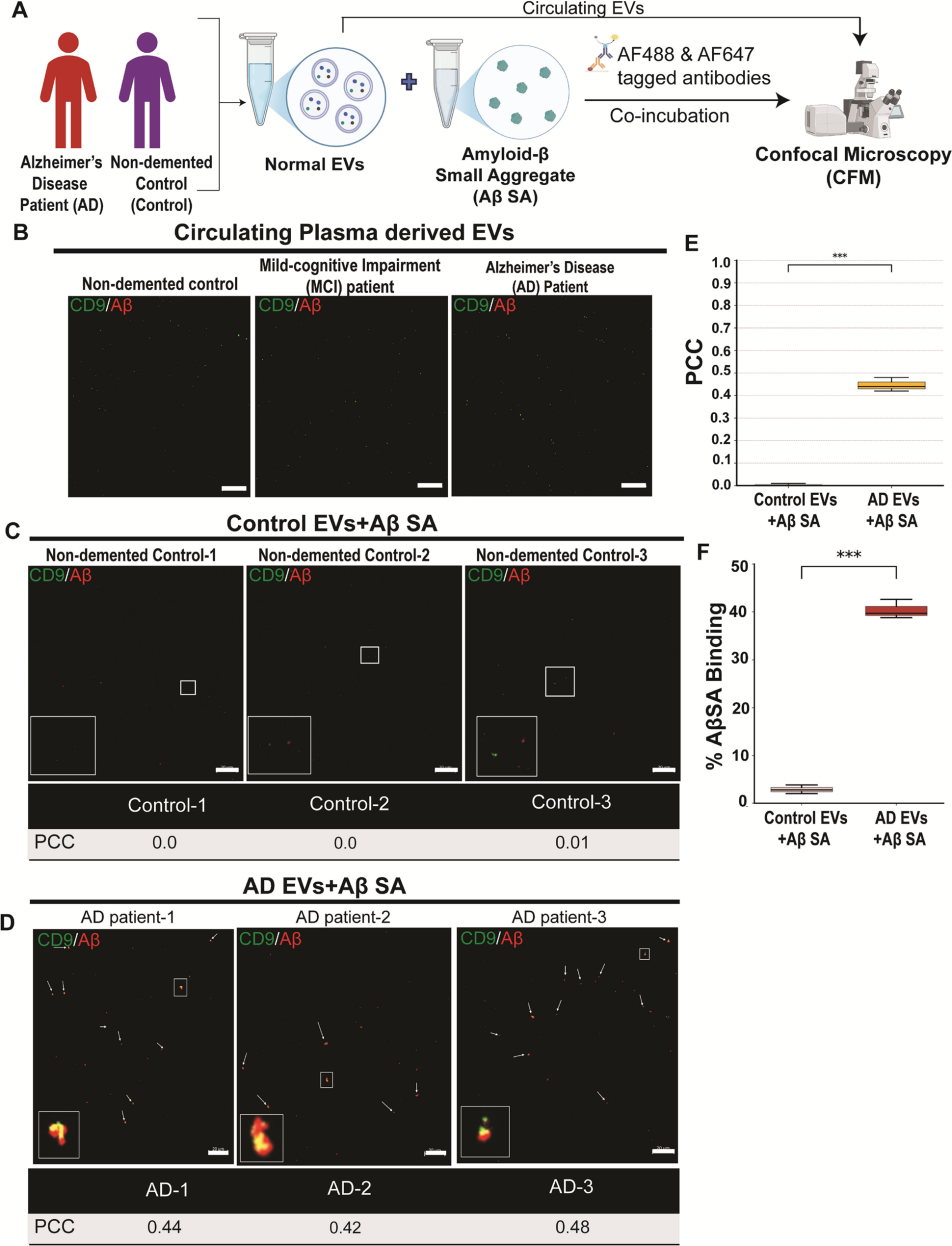
Graphical representation of workflow **A**. Circulating sEVs derived from Control, MCI and AD patient does not show colocalization signal **B**. Co-incubation with Aβ SA was performed only for control and AD EVs. Controls sEVs incubated with small amyloid-β aggregates **C**. AD sEVs, when incubated with small amyloid-β aggregates, colocalize (White arrows) at low temperature 4℃ **D**. Colour Yellow is the colocalised signal for sEVs (Green)and Aβ (Red). Scale bar= 20 μm. PCC (**E**) between CD9–Alexa Fluor 488 EVs and AβSA-Alexa Fluor 647, calculated using Costes thresholding (FIJI/JACoP). Control EVs: 0.033 ± 0.005; AD EVs: 0.780 ± 0.046 (*n* = 3). One-way ANOVA: *p* < 0.0001. Percentage of AβSA associated with EVs (**F**), Control EVs: 2.87 ± 0.90%; AD EVs: 40.37 ± 1.98% ((*n* = 3; incubation maintained at 4 °C)). Unpaired t-test: *p* < 0.0001. AD (*n* = 9), MCI (*n* = 3), and non-demented controls (*n* = 10)

Having determined that altered sEVs preferentially sequester small amyloid-β aggregates, we investigated this association in circulating fluids, such as plasma, to assess whether these in vitro coaggregates can be detected. We isolated circulating EVs from non-demented controls (NDC), and patients with mild cognitive impairment (MCI) and Alzheimer’s disease (AD), then incubated them with Anti-CD9 and Anti-Amyloidβ42 antibodies (Fig. 10A). Baseline CD9-Aβ colocalization was assessed in circulating EVs from age-matched controls, MCI, and AD patients with no detectable association observed. Co-incubation with small Aβ aggregates (SA) was performed for control and AD. The Confocal image showed no association between the two signals in circulating EVs, in contrast to our in-vitro findings (Fig. 10B, Supplementary Fig. 8A). This is likely because extracellular amyloid-β aggregates cannot transverse the Blood-brain barrier (BBB) in association with circulating EVs. Next, we incubated sEVs from Healthy controls with SA but observed no association (Fig. 10C, Supplementary Fig. 8B). However, when AD EVs were co-incubated with SA, confocal images showed association between the two signals in AD patient samples corroborating with the in-vitro findings (Fig. 10D, Supplementary Fig. 8C). PCC value for AD EVs co-incubated with SA was significantly higher (*p* < 0.0001) compared to control EVs co-incubated with SA (Fig. 10E). Also, the percentage of AβSA associated with EVs was significantly higher (*P* < 0.0001) for AD EVs that were co-incubated with SA. This association may be due to the oxidative stress-induced conformational change in the sEVs membrane, making them more likely to associate with the Aβ SA aggregates.

## Discussion

### EVs biological relevance

Cell-to-cell communication is mediated through various mechanisms, with extracellular vesicles playing a key role in the exchange of biomolecules secreted by cells. EVs are found in all biofluids and carry molecular signatures reflective of their cell of origin, which can influence various cellular processes under normal and pathological conditions [36]. In this study, sEVs were isolated using a chemical precipitation method followed by ultrafiltration and size-exclusion chromatography yielding analytical-grade sEVs, consistent with the results observed in our previous studies [37–39]. We assessed EVs size distribution and particle concentration by NTA. Additionally, we used the immunoblot to examine the expression of surface markers such as CD9, CD63 and CD81 and the presence of specific luminal markers like Tsg101.

### Amyloid-β (Aβ) and sEVs in Alzheimer’s disease (AD)

Amyloid-β aggregates were prepared following the protocol by Lim et al. (2019) [13] to model species relevant to Alzheimer’s disease (AD) pathology. Different-sized aggregates of Aβ-42 were generated, and their structural morphology and size distribution were confirmed through TEM, CFM, DLS, and Thioflavin-T (ThT) fluorescence. EVs are known to carry amyloid-β (Aβ) as cargo and contribute to the seeding effect in Alzheimer’s disease (AD) progression [15]. Previous studies have observed that Aβ aggregates can bind to EVs membranes through Transmission electron microscopy (TEM) [13]. These studies have reported TEM-based examination of EV-Aβ interactions by immobilization on grids. However, there is a need to study these interactions in a physiological-like state, as we have observed alterations in sEVs membrane in response to external environmental conditions. Furthermore, to investigate the interaction between EVs and Aβ, we employed an immune-fluorescence strategy followed by their visualization using confocal microscopy (CFM) and TEM. Both TEM and CFM revealed that SA exhibited the highest binding affinity to EVs, and quantitative analysis, such as PCC, revealed maximum colocalization for SA group. PCC values for SA were 0.88 compared to minimal association with UA (PCC = 0.07) and BA (PCC = 0.02). This demonstrated that SA preferentially binds to sEVs compared to other Aβ aggregates. It was also clear from our experiment that under normal conditions, EVs do not bind Aβ aggregates. We used EV concentrations of ∼10^9 particles/mL and Aβ [1–40] monomer concentration of 20 µM as reported earlier by Halipi et al. 2024 who have extensively studied the intersection between EVs and Aβ [1–40] peptides [16]. We performed the temporal assessment of EVs-Aβ co-incubation for 24, 48, and 72 h at both physiological temperature 37 °C and low temperature 4 °C to account for temperature-induced effects. EVs-Aβ co-incubation experiments at 37 °C revealed a time-dependent increase in Aβ aggregate size, accompanied by EV aggregation confirming that physical conditions have a significant impact on the sEVs membrane [31, 32]. This alteration of the EV membrane scaffold impacts the affinity to Aβ resulting in its sequestration whereas no major differences were observed in the experimental group at 4℃. Interestingly, subjecting EVs to mechanical stress such as ultrasonication before incubation at 4 °C increased EVs association with Aβ. These findings indicate that alteration to the EVs membrane by mechanical (Ultrasonication and agitation) and physical (temperature) stress plays a critical role in Aβ sequestration.

### Effect of oxidative stress on EVs and mechanisms of Aβ sequestration by EVs and its role in disease progression

We investigated whether oxidative stress, a well-recognized factor in Alzheimer’s disease (AD) pathogenesis, alters EVs thereby influencing Aβ sequestration. To model this, we subjected EVs to a minimal dose of oxidative stress inducer i.e.,1 µM H_2_O_2_, for 24 h and compared them to ultrasonicated EVs. Both treatments led to comparable structural distortions in sEVs, as observed by transmission electron microscopy (TEM) and confocal microscopy, this was observed even at lower temperature conditions i.e., 4 °C. These findings were consistent across temporally assessed groups- 24, 48, and 72 h whereby a gradual increase in the colocalization coefficient (PCC) indicating association of EVs-Aβ signals was observed. This implies active sequestration by the altered EVs over time. We also observed that the structural morphology of EVs was distorted in both groups compared to control EVs.

In addition to this, insight from lipidomic analysis of AD patients’ sEVs demonstrated elevated levels of oxidized glycerophospholipid, which supports our inference that oxidative stress alters EVs membrane scaffolds, influencing amyloid-β sequestration. Our results demonstrated that both oxidative stresses induced by H_2_O_2_ and mechanical (Ultrasonication) stress alter EVs membrane scaffold enhancing their affinity to Aβ which results in its sequestration. Untargeted lipidomic analysis revealed that lipid classes such as lysophosphatidylcholine (LPC), sphinganine (SPB), and ceramide (Cer) were highly dysregulated in stress-altered EVs compared to normal sEVs, and lipid levels in the AD group closely mirrored those observed in sEVs exposed to oxidative stress. Notably, studies show that Lysophosphatidylcholine (LPC) is elevated in oxidatively damaged tissues, and is also well-known for its role in the oligomer formation process of Aβ1–42 peptide, leading to a subsequent cascade of apoptosis and neuronal death [40–42]. In our study, the small aggregates which represent oligomers, are preferentially sequestered by altered sEVs enriched in these lipid classes such as LPC, SPB, which in turn promote EV-Aβ coaggregates that are subsequently internalized by cells, contributing to pathogenesis. This causes selective sequestration by altered sEVs, highlighting their affinity for small aggregates (SA). Furthermore, SA may disrupt lipid membranes and internalize into neuronal cells, mediating toxicity. This process is likely to exacerbate neurodegeneration by triggering neuroinflammation and apoptotic pathway cascades thereby amplifying neuronal damage.

This study also distinguishes between small aggregates (SA), which are preferentially sequestered by EVs, and larger aggregates (BA), which exhibit minimal interaction. This differentiation addresses the heterogeneity reported in TEM-based studies on EV-Aβ interactions. By studying these interactions in solution using an immunofluorescence approach and confocal microscopy, we avoided artifacts caused by immobilization on grids. Another study [13] have reported the prefibrillar Aβ aggregates binding preferentially with exosomes, however, the higher temperature conditions used in the study itself could be the driving force behind the EV-Aβ association due to sEVs membrane alteration. This is due to the fact that structural flexibility and exposure of hydrophobic surfaces are critical factors that determine the capacity of oligomeric assemblies to cause cellular dysfunction, potentially resulting in outcomes such as neurodegeneration [43]. A recent study reports that tau is tethered to the EVs luminal membrane [44] as observed in the Cryo-EM imaging, inviting clarification behind this interaction as it leaves questions about the presence of tethering sites at EVs membrane, as tethering may require an adaptor or direct anchoring. Based on our observations, we propose that oxidative stress-induced conformational changes in EVs expose the limiting membrane, creating tethering sites for small aggregates. Studies have consistently shown that oligomers can interact with and permeabilize both synthetic lipid vesicles and cell membranes [45]. Their high structural plasticity and hydrophobic surfaces enable oligomeric aggregates to penetrate cell membranes, traverse them, and reach the cell interior [46]. Studies have shown that soluble oligomers from various types of amyloids increase lipid bilayer conductance, irrespective of their sequence. In contrast, fibrils and low molecular weight soluble species do not produce this effect. Notably, this increase in membrane conductance occurs without clear evidence of channel or pore formation or any ion selectivity [47].

Furthermore, we also observed an enrichment of EVs CD9 marker around amyloid-β plaques where CD9 signals were localized around amyloid plaques in human AD and transgenic APP-PS1 mouse brains. This is consistent with previous findings showing the presence of exosomal proteins, such as flotillin-1 and Alix, near these plaques. Previous studies have reported that flotillin-1 labeled was most concentrated in areas containing senile plaques in the cerebral cortex, while the amyloid core of mature plaques was mostly negative for flotillin-1 [48] and an accumulation and enrichment of Alix were observed around amyloid plaques in AD patient’s brains [12].

We also examined the circulating sEVs from non-demented controls (NDC), mild cognitive impairment (MCI), and Alzheimer’s disease (AD) for EVs and amyloid-β association. No association was observed, likely because extracellular amyloid-β aggregates cannot traverse the blood-brain barrier (BBB). Similarly, EVs from healthy controls did not show an association when incubated with SA; in contrast, a significant association was observed when sEVs from AD patients were incubated with SA, possibly due to a subset of altered EVs in circulation or disease-specific changes in EVs.

### Conclusions and implications for Alzheimer’s disease progression

**Fig. 11:**
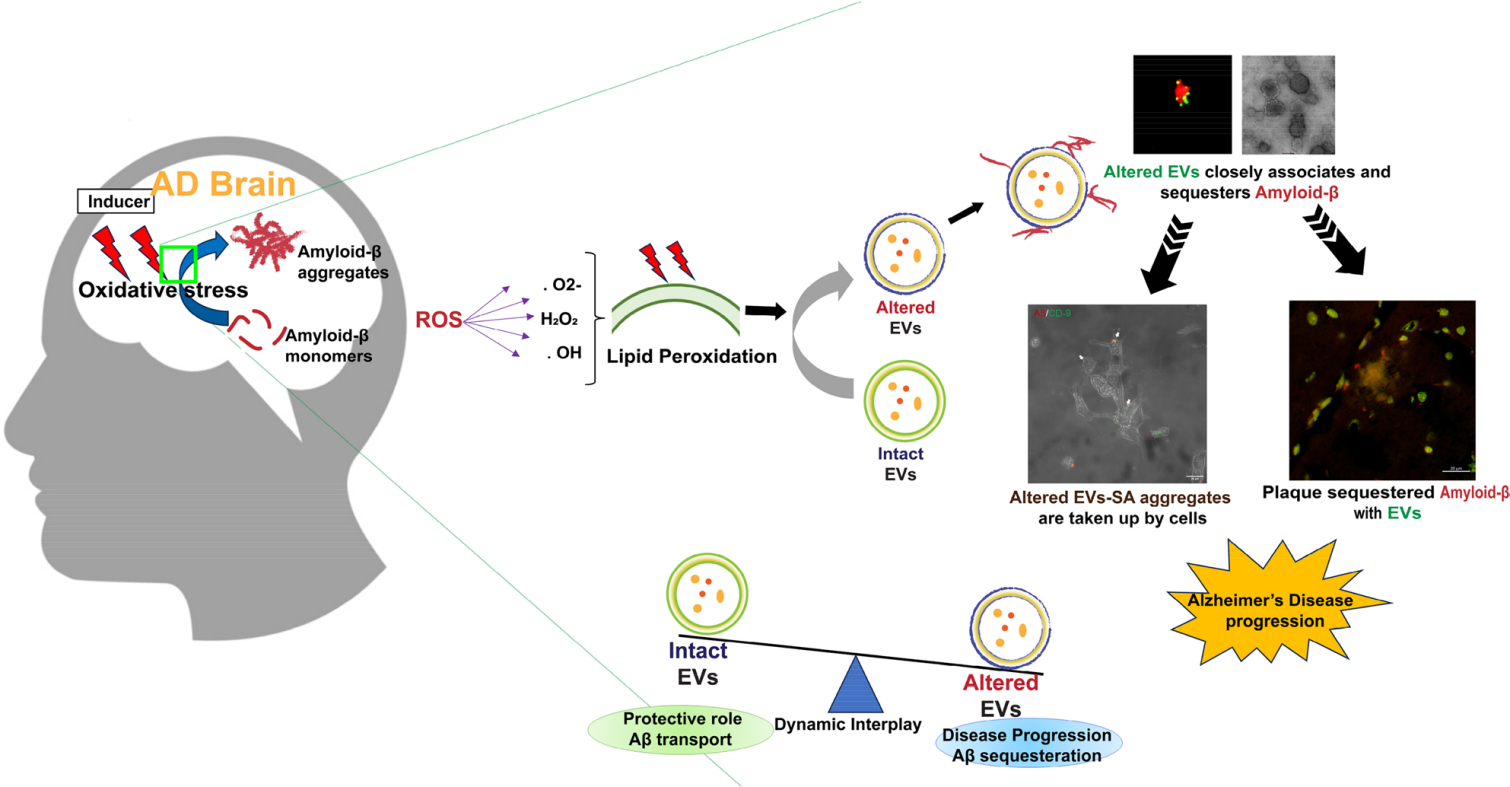
Mechanisms of Aβ sequestration by stress-altered sEVs and their implication in disease progression

In conclusion, higher temperature conditions [31, 32], H_2_O_2_- [49, 50] and ultrasonication treatments led to significant distortion and self-aggregation of EVs. Structural changes in EVs are due to exposure to these external stresses that alter EVs membrane scaffold, which facilitates the sequestration of extracellular Aβ. We also validated the role of oxidative stress, driven by reactive oxygen species, as a key factor in Alzheimer’s disease (AD) pathogenesis, altering EVs membrane conformation and influencing Aβ aggregation. Notably, the strength of our study stems from a clear demarcation of Aβ species and small aggregates (SA) preferentially sequestered by EVs. Our finding is limited by the challenge of detecting EVs-Aβ coaggregates in patient samples due to blood-brain barrier (BBB) selectivity. However, sEVs from AD patients co-incubated with small Aβ aggregates (SA) exhibited a clear association, thus corroborating our in vitro results. This association likely stems from oxidative stress-induced alterations in EV membranes that enhance their affinity for Aβ SA. In summary, our study highlights the coveted role of EVs in AD progression. While EVs are typically known to support neuroprotection by clearing Aβ, altered sEVs sequester Aβ and aid in plaque formation, and altered EV-Aβ coaggregates traverse neuronal membranes to mediate toxicity, thereby driving disease progression (Fig. 11). Our study provides a novel insight into the oxidative stress-mediated EV membrane conformational changes and their relevance in AD disease progression.

## Materials & methods

### Sample collection

The study included non-demented controls with no family history of cancer, autoimmune diseases, neurodegenerative disorders, or amyloidosis, as well as Alzheimer’s disease patients diagnosed through neuropsychological assessments (ACE-III and MMSE) conducted by certified geriatric practitioners (Supplementary Table 1). Ethical approval for the study was granted under IECPG-213/20.04.2023, and all participants were enrolled after providing signed 9informed consent. Five millilitres of blood were collected from each participant via venipuncture into EDTA vials. Post-centrifugation of 2500 g for 20 min at 4 °C to pellet down the cells, plasma was collected, and subjected to centrifugation for 30 min at 4 °C and 10,000 g. Cleared plasma was aliquoted and stored at -80 °C. Post storage, the samples were centrifuged at 10,000 g after being thawed on ice and used for the downstream experiment.

### Isolation of sEVs

Small Extracellular vesicles (sEVs) were isolated using size exclusion chromatography (SEC) columns (Izon qEV1 70 nm Gen 2). We also isolated sEVs using the lab-developed filtration with precipitation method as described in [51]. 1 ml of clarified plasma sample was used for isolating sEVs from SEC columns. 1X PBS (ML116-500ML, HiMedia) was used to clean the column The fraction number (F) 1–5 enriched in sEVs was subsequently used for downstream experiments.

### Aβ42 aggregate formation

Aβ42 peptides (β-Amyloid [1–40] 1932-2-15) were purchased from DGpeptides Co., Ltd. 1 mg of peptide was dissolved in 1 mL of Hexafluoroisopropanol (HFIP) in a clean vial, then vortexed until fully dissolved. The resulting solution was aliquoted into 10 MCTs and sealed before flushing with nitrogen to evaporate the HFIP completely. After evaporation, the peptide was lyophilized before proceeding with any further processing. Different amyloid-β aggregates were prepared according to *Lim. et al. 2019* [13]. Aβ42 peptides were resuspended in 60 mM NaOH and vortexed rigorously. To maintain the peptides at physiological pH, osmolarity, and ion concentration, the Aβ42 peptides were diluted to 20 µM, Un-aggregated Aβ42 (Aβ42 UA), pH 7.4 in 1X PBS Solution (Himedia, ML116). For making Aβ42 Small Aggregates (Aβ42 SA), 20 µM Aβ42 peptides, pH 7.4 were ultrasonicated using a benchtop bath sonicator at 50 kHz and 80 W for 5 min. For Aβ42 Big Aggregates (Aβ42 BA) preparation, after sonication step, it was incubated at 37 °C with rapid shaking for 2 h. Aβ42 preparations were flash-frozen in liquid nitrogen and stored at − 80 °C.

### α-synuclein aggregate formation

Recombinant human alpha-Synuclein protein (Met1–Ala140), expressed in *E. coli* (Cat# SP-485-500, R&D Systems), was used to induce aggregation following a modified protocol [52]. A 20 µM working solution was prepared from a 60 µM stock in 10 mM Tris-Cl buffer (pH 7.4) containing 1 mM EDTA and 10 mM NaCl. The mixture was incubated at 37 °C at 250 rpm in the presence of glass beads for seven days to promote fibril formation. Aggregation was confirmed using Transmission Electron Microscopy (TEM).

### Thioflavin T (ThT) assay

Thioflavin T (ThT), Sigma was dissolved in phosphate-buffered saline (PBS) and filtered using a 0.2 μm syringe filter. ThT was then added to 20 µM Aβ42 UA, Aβ42 SA, and Aβ42 BA solutions, respectively, to achieve a final ThT concentration of 60 µM. The samples were incubated at 37 °C for 2 h. Following incubation, 40 µL of each ThT sample was transferred to a black 384-well opaque bottom plate. ThT fluorescence was measured at room temperature using a Spectramax i3x plate reader with an excitation wavelength of 450 nm and an emission range of 480 to 600 nm.

### Western blot

All samples were normalized according to the initial volume of biofluid input, i.e. 180 µl [38, 39]. Then, 20 µL was loaded to run on an 8–12% SDS PAGE after the sEVs sample and the sample loading dye (2 × Laemmle Sample buffer) were combined. Following SDS-PAGE, the protein from the gel was wet transferred onto a 0.22 μm PVDF membrane (1,620,177, BioRad). Membrane was blocked with 3% bovine serum albumin (BSA) (D0024, BioBasic) in Tris (TB0194, BioBasic) base saline containing 0.1% of Tween 20 (65,296, SRL Chem) (TBST). Blot was incubated with primary antibodies against CD63 (10628D, Invitrogen), CD81 (PA5-86,534, Invitrogen), TSG101 (MA1-23,296, Invitrogen) and CD9 (PA5-86534, Invitrogen) were incubated at 4 °C for the entire night. Before being incubated at room temperature with HRP-conjugated secondary antibodies, anti-rabbit (AB6721, Abcam), and anti-mouse (31,430, Invitrogen), the membranes were washed three times with TBST buffer. Apolipoprotein levels were also assessed to check the level of protein co-isolates.The blot was developed using the Femto LUCENT™ PLUS-HRP kit (AD0023, GBiosciences) to visualize the protein bands through enhanced chemiluminescence.

### Nanoparticle tracking analysis (NTA)

The NTA of sEVs was performed after 5000-fold dilution in 1X-PBS buffer. One milliliter of a diluted sEVs sample was added to the sample chamber of the ZetaView Twin system (Particle Metrix, Germany). The following settings were applied during three cycles of scanning eleven different cell locations: high video setting, autofocus: focus, shutter: 150, camera sensitivity: 80, and cell temperature 25 °C. A total of sixty frames were gathered for each position. The built-in ZetaView Software 8.05.12 (Particle Metrix, Germany) was used for analysis, with CMOS cameras for recording. The least particle size was 10 nm, the maximum particle size was 1000 nm, and the minimum particle brightness was 30.

### Dynamic light scattering (DLS)

1 µl of Aβ42 preparations (Aβ42 UA, Aβ42 SA, Aβ42 BA) was added to 1000 µl of 1XPBS, vortexed, and transferred to a precleaned sample cuvette. Hydrodynamic size (Rh) distribution measurements were performed by a zeta particle size analyzer (Malvern).

### Co-incubation of sEVs with different Aβ42 preparations

#### Assessment of EVs -amyloid beta association

Approximately 9.0 × 10^8^ particles of sEVs were introduced to 20 µM Aβ42 UA solution. The mixture was subjected to sonication using a benchtop sonicator at 80 W power and 60 kHz frequency. Following sonication, the samples were incubated for 2 h with agitation.

#### Evaluation of sEVs’ affinity with different amyloid-beta aggregates

Aβ42 UA, Aβ42 SA, and Aβ42 BA, each at a concentration of 20 µM, were individually combined with approximately 6.0 × 10^8^ particles of sEVs. The mixtures were incubated for 2 h at 25 °C. Imaging was performed following the incubation period.

#### Assessment of amyloid beta-sEVs association in a time-dependent manner

Aβ42 UA was incubated with ~ 10^9^ EV particles at room temperature and 4 °C. The association between Aβ42 UA and sEVs was assessed by imaging at different time points, specifically after 24, 48, and 72 h of incubation.

#### Amyloid beta association with ultrasonicated sEVs

A 20 µM solution of Aβ42 SA was added to approximately 9.0 × 10^8^ EV particles pretreated by ultrasonication using a benchtop sonicator. The combined sample was then incubated at 4 °C, and imaging was performed at 24, 48, and 72-hour time points to assess the interaction between Aβ42 SA and the sonication-induced disrupted sEVs.

#### Amyloid beta association with sEVs in the presence of H_2_O_2_

~ 9.0 × 10^8^ EV particles were combined with 20 µM Aβ42 SA in the presence of 100 µM hydrogen peroxide (H₂O₂). The mixture was incubated at 4 °C and imaged at two-time points: immediately (0 h) and after 24 h.

### Transmission electron microscopy

The ultrastructural morphology of sEVs was studied using transmission electron microscopy. The isolated sEVs were diluted (1:1000) with filtered 1× PBS. Similarly, for the characterization of different Aβ42 peptides and aggregates, 20 µM solution of Aβ42 UA, Aβ42 SA, and Aβ42 BA were diluted to 1000-fold in filtered 1× PBS. Finally, sEVs and Aβ42 preparations were co-incubated and the samples were diluted 2500X in 1X PBS. Subsequently, small drops (50 µl) of the diluted sample suspension were adsorbed at room temperature for 15 min using a 300-mesh carbon-coated copper grid (01843, Ted Pella). Following this, grids were gently dabbed to remove excess liquid and then positioned onto a 20 µL droplet of filtered MilliQ water. As a negative stain, 2% aqueous uranyl acetate solution (81,405, SRL Chem) was used for 10 s. The grids were blot-dried and examined with a Thermo Scientific Talos S transmission electron microscope.

### Confocal microscopy

#### Characterization of sEVs

20 µl of isolated sEVs were incubated for 2 h at 25 °C with 0.4 µl of Human CD9 Alexa Fluor^®^ 488-conjugated antibody (R&D systems, FAB1880G) and imaged using a Zeiss LSM980 confocal microscope (Carl Zeiss Microscopy).

#### sEVs and Aβ42 preparations co-incubated

sEVs and Aβ42 suspension was labeled using Human CD9 Alexa Fluor^®^ 488-conjugated Antibody (R&D systems, FAB1880G) for sEVs and Alexa Fluor^®^ 647 Anti-beta Amyloid 1–42 antibody [mOC64] (Abcam, ab300742) in final concentration of 2%(v/v) and 0.5%(v/v) of the total suspension respectively. sEVs and the aggregate suspension were incubated for 2 h with both antibodies at 25 °C before mounting on labolene-cleaned glass slides (Blue Star) and covered with 18 mm;10Gms glass covers (Blue Star), and were imaged on Zeiss LSM980 system using the 40X objective. The acquisition parameters were kept constant for the same set of experiments.

### dSTORM microscopy

STORM images were acquired using a custom-built setup with a Nikon Ti2E microscope body. Briefly, Aβ42 labelled with Alexa Fluor^®^ 647 Anti-beta Amyloid 1–42 antibody [mOC64] (Abcam, ab300742) was excited with a 647 laser (MPB Communications). Upon excitation, the emitted light was collected by Nikon 100x oil immersion TIRF-SR objective (NA 1.49) and a laser quad band set with emission filter (TRF89902-EMET-405/488/561/647 nm laser Quad Band Set for TIRF application; Chroma). An EM-CCD camera was used for capturing images and hence, image localization, at an exposure time of 20 milliseconds per frame for 40,000 frames for each STORM image. Reconstruction of the image was done using custom-written software (Insight3, provided by B. Huang, University of California, San Francisco, CA, USA) [53].

To understand the degree of interaction with SEC-isolated sEVs labeled using Human CD9 Alexa Fluor^®^ 488-conjugated Antibody, Total Illumination Reflection Microscopy was performed on the same sample, using the same microscope. The TIRF images were captured with the EM-CCD camera at an exposure time of 100 milliseconds per frame for 10 frames. Upon acquisition, the TIRF images were overlaid on the STORM images with the image coordinates in place to study the interaction between the aggregate and the extracellular vesicles.

### Lipidomic study

Lipids were isolated using modified Folch method which employed a biphasic solvent system composed of chloroform and methanol in a 2:1(v/v) ratio [54]. The LC-MS analysis was performed on a high-resolution Orbitrap Exploris 120 mass spectrometer (Thermo Fisher Scientific) equipped with a Vanquish UHPLC (Thermo Fisher Scientific). The acquired data was processed using Compound Discover software (3.2.1 version; Thermo Fisher Scientific), and metabolite screening was done as an untargeted approach.

### Cell-internalization of EV-Aβ coaggregates

SH-SY5Y human neuroblastoma cells were grown in DMEM/F-12 (Dulbecco’s Modified Eagle Medium/Nutrient Mixture F-12), Gibco™ (Cat: 11320033), supplemented with 10% qualified fetal bovine serum (FBS), Gibco™ (Cat: 10270106), and 1% Antibiotic-Antimycotic (100X), Cat: 15,240,062, under standard conditions at 37 °C in a humidified atmosphere containing 5% CO₂. Cells seeded on Nunc™ Glass Bottom Dishes Cat: 150,680 and were first treated for 60 min at 37 °C with EVs alone, Aβ aggregates alone, and EV-Aβ coaggregates. A total of 3 µg protein was used. EVs and Aβ were labeled with primary antibodies conjugated with fluorophore namely, AlexaFluor647 anti-amyloidβ and AlexaFluor488 anti-CD9 antibodies. Spent media was replaced with fresh media before imaging.

### Brain tissue staining protocol

APP mice brain tissue sections and autopsy brain tissue sections were previously used in a study [55]. Brain specimens were post-fixed in 4% paraformaldehyde for 72 h, then transferred to 15% sucrose in PBS (Cat # S5-3, Fisher Scientific) until tissue settled to the bottom. Subsequently, tissues were moved to 30% sucrose in PBS until they sank to the bottom, indicating complete saturation. The tissues were embedded in an OCT compound and sectioned at a thickness of 12–15 μm using a cryotome. Cryo-sectioned brain slices were obtained from 13-month-old APPswe-PSEN1 mice (male) and age-matched wild-type (WT) mice (male). Brain slices were deparaffinized in xylene and rehydrated through sequential washes in 90%, 75%, 50%, and 30% ethanol, followed by a final rinse in 1× PBS, with each step lasting 5 min. EVs were labeled using a primary CD9 antibody (Catalog # PA5-86534, Invitrogen), followed by a TRITC-conjugated secondary antibody (Goat anti-Rabbit IgG (H + L), Catalog # A16101). Amyloid-beta (Aβ) deposits in the brain tissue sections were stained with 0.1% aqueous ThT solution. The sections were incubated for 60 min and then washed three times with PBS, 5 min per wash. Prepared tissue slices were mounted and visualized using a laser confocal microscope (LSM980, Carl Zeiss) at 40× magnification.

### Colocalization and statistical analysis

All confocal images were processed using the Zen 3.9 image processing software. Small extracellular vesicles (sEVs) labeled with Human CD9 Alexa Fluor^®^ 488-conjugated antibody were visualized in the green channel. In contrast, amyloid-β aggregates, labeled with Monoclonal Alexa Fluor^®^ 647 anti-beta amyloid 1–42 antibody (Abcam, ab224025), were observed in the red channel. Colocalized signals appeared yellow. In addition to visual colocalization, pixel-by-pixel colocalization analysis was performed using Zen 3.9. The intensity of the one-color channel was plotted against the intensity of the second color for each pixel in a scatterplot to graphically represent colocalization. Background noise was removed by applying threshold values determined by the Costes method [56]. Colocalization was quantitatively assessed using Pearson’s Correlation Coefficient (PCC), ranging from + 1 (indicating perfectly linearly related intensities) to -1 (indicating perfectly inversely related intensities), and was also used to quantify the strength of the correlation between the fluorescence intensities of the two channels [35, 57].

## Supplementary Information

Supplementary Material 1.

Supplementary Material 2.

Supplementary Material 3.

Supplementary Material 4.

Supplementary Material 5.

Supplementary Material 6.

## Data Availability

No datasets were generated or analysed during the current study.
