# Supplementary material for: Stress-induced alteration of small extracellular vesicles drives amyloid-beta sequestration and exacerbates Alzheimer’s disease pathogenesis
Source: Alzheimers Res Ther. 2026 Apr 11;18:84. doi: 10.1186/s13195-026-02028-1 (PMC13072553; doi:10.1186/s13195-026-02028-1)
Supplement: Supplementary file 1 — Supplementary Material 1. [file 13195_2026_2028_MOESM1_ESM.docx]

**
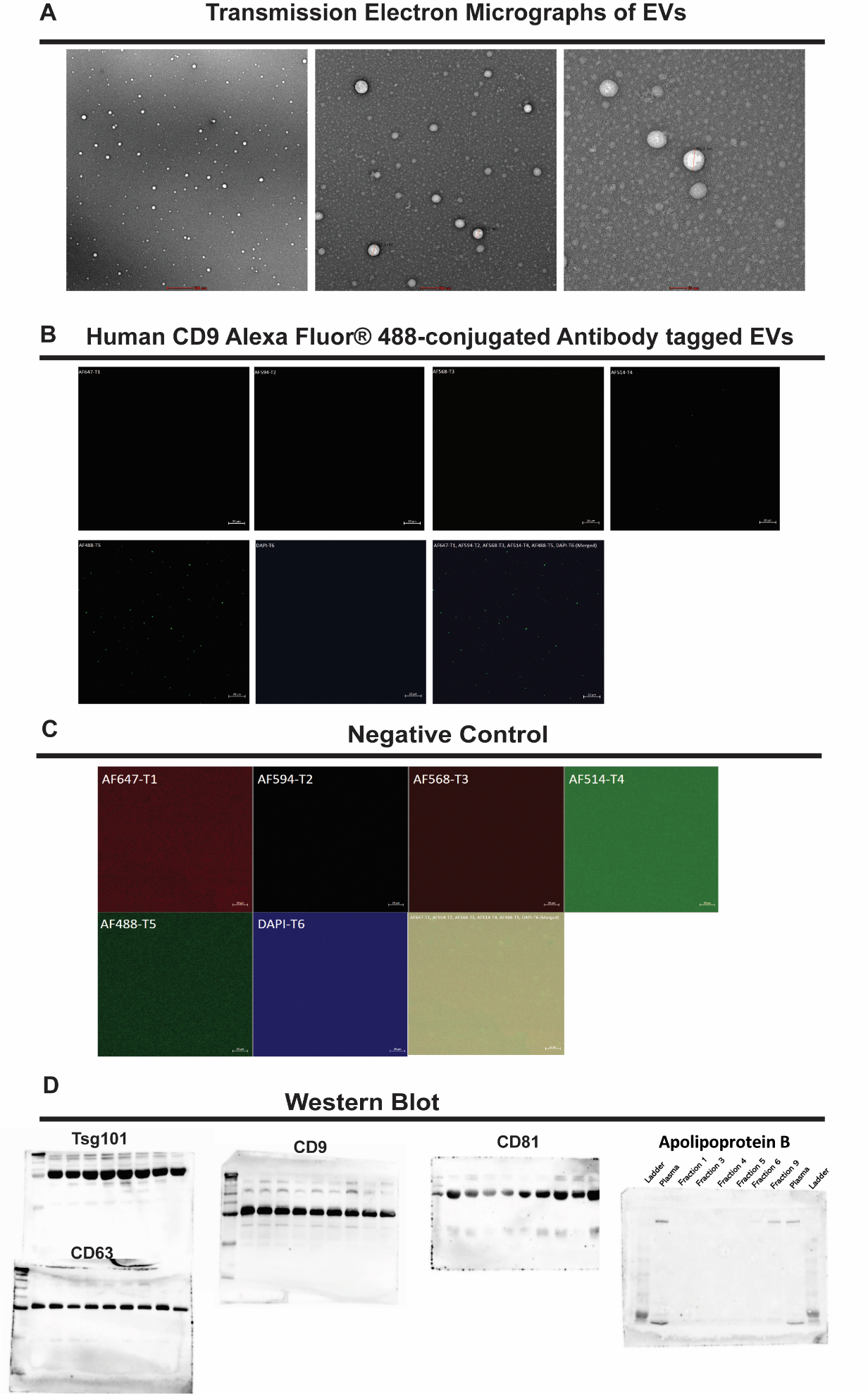
Supplementary Figure 1:** TEM images of plasma-derived EVs (A); CFM Split image showing Alexa-Fluor-CD9 signals in all channels (B) and in negative control (PBS) (C); Western blot (Full blot) for EVs marker proteins and co isolated protein (Apo-B)(D).

**
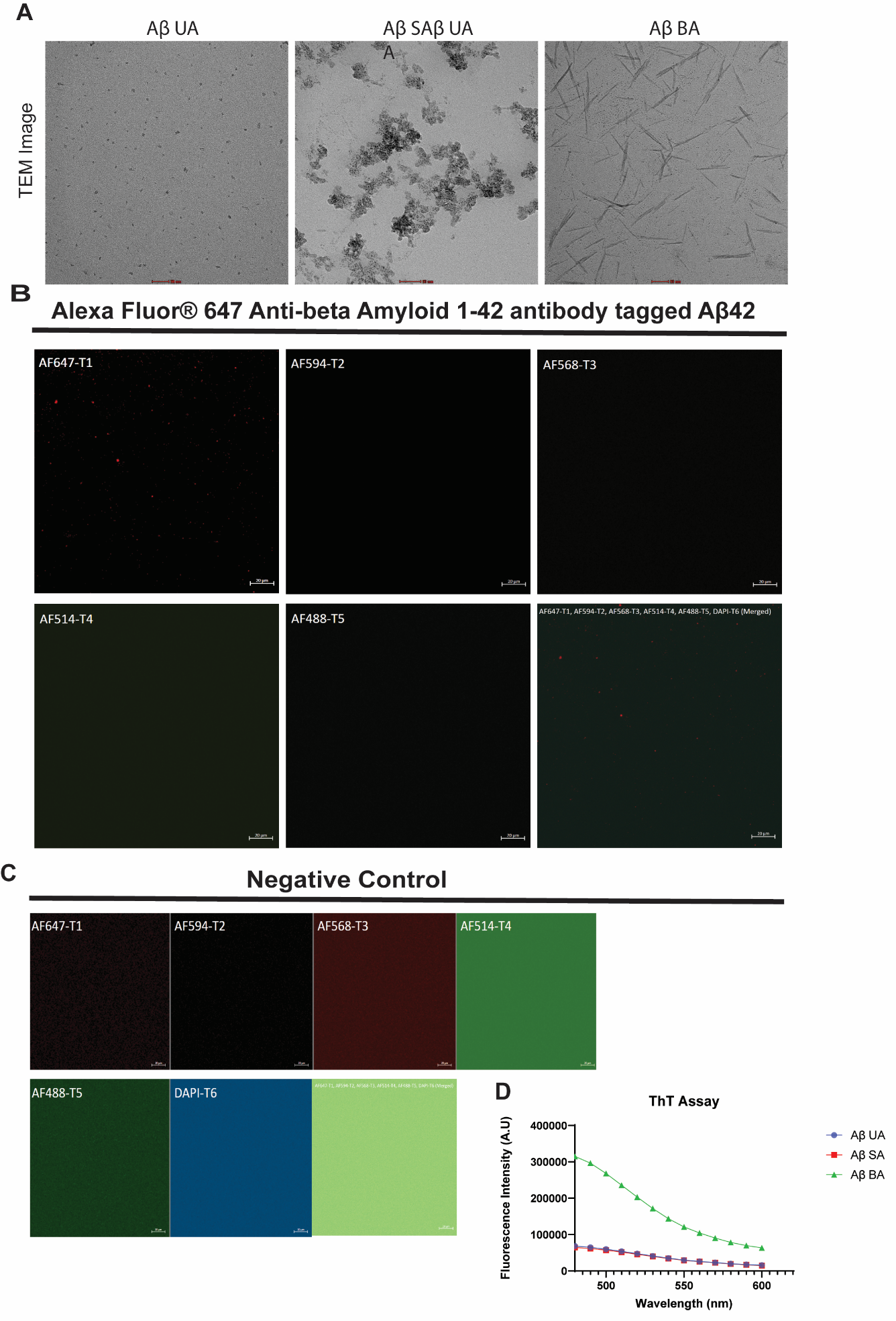
Supplementary Figure 2:** TEM images of different Amyloid-β aggregates(A); CFM Split image showing Alexa-Fluor-647 Amyloid-β signals in all channels (B)and in negative control (PBS) (C); ThT Fluorescence for three Aβ groups (D).

**
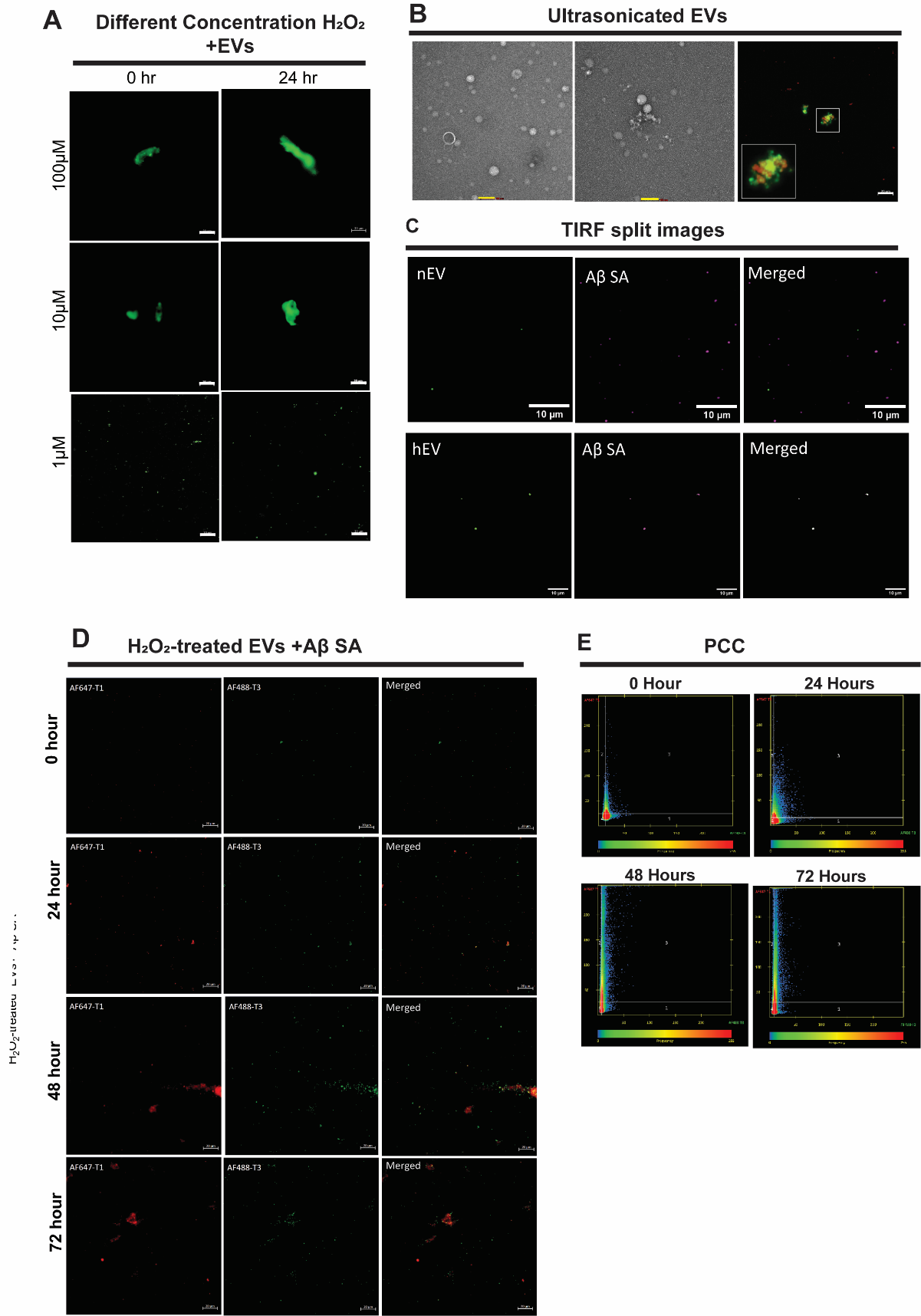
**

**Supplementary Figure 3:** Confocal image of EVs treated with different concentration of H_2_O_2_(A), Ultrasonicated EVs(B) , TIRF split images showing Alexa-Fluor-488 CD9 (Green) and Alexa-Fluor-647 Amyloid-β (Magenta) signals of: normal EVs+ Aβ(SA) & H_2_O_2_-treated EVs + AβSA. Colour white is the merged signal of EVs (Green) and Aβ (Magenta) (C), H_2_O_2_-treated EVs+ AβSA at 0-72hrs at 4℃. Colour yellow is the merged signal of EVs (Green) and Aβ (Red)(D)Scale bar= 20μm.(C) PCC graphs for 0,24,48 and 72 hours) (D).


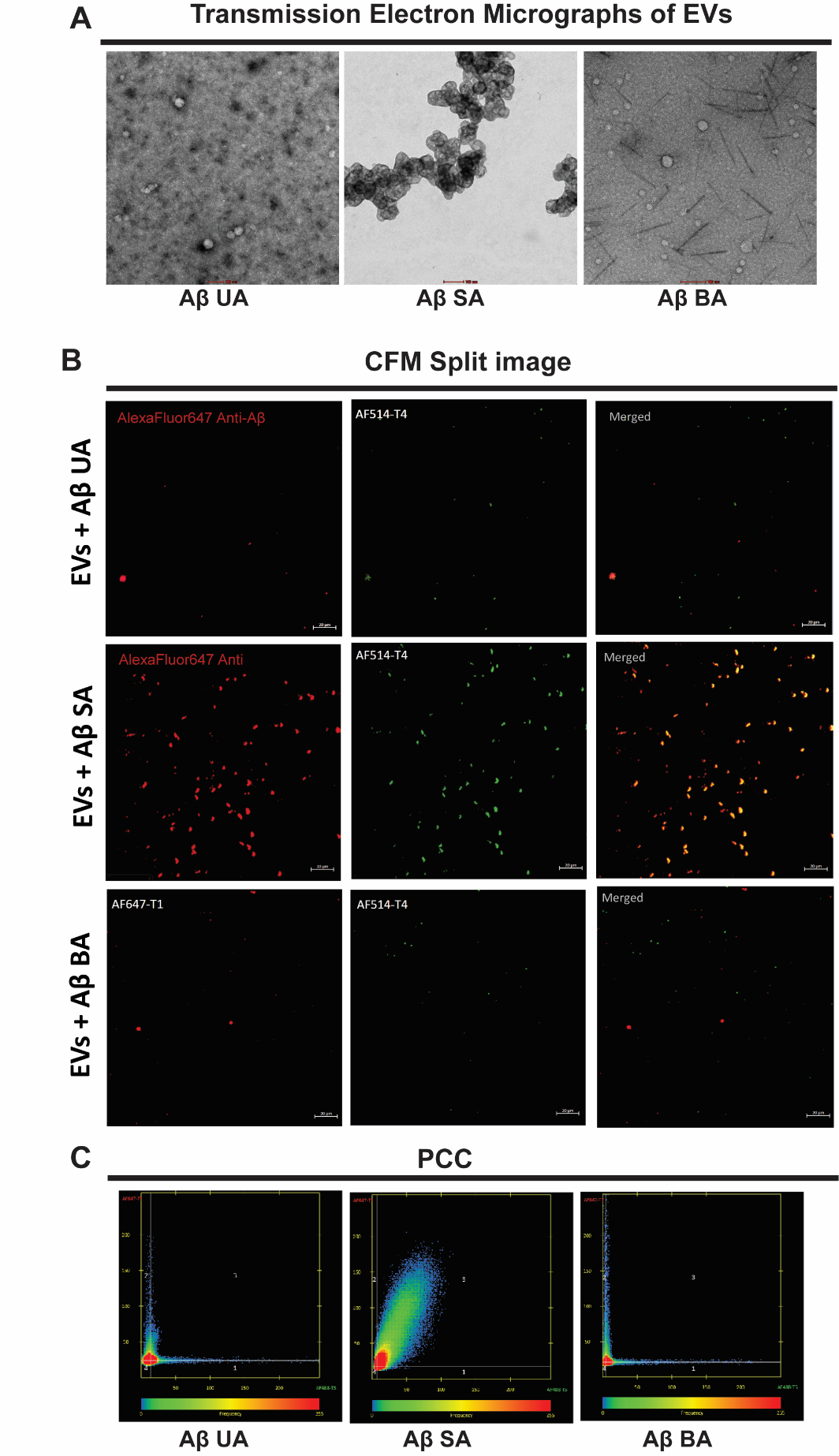


**Supplementary Figure 4:** TEM images of EVs and Aβ UA, SA and BA. Scale bar 50nm. (A); CFM Split image showing Alexa-Fluor-488 CD9 and Alexa-Fluor-647 Amyloid-β signals for all 3 groups. Colour yellow is the merged signal of EVs (Green) and Aβ (red). Scale bar= 20μm. PCC graph (C) .


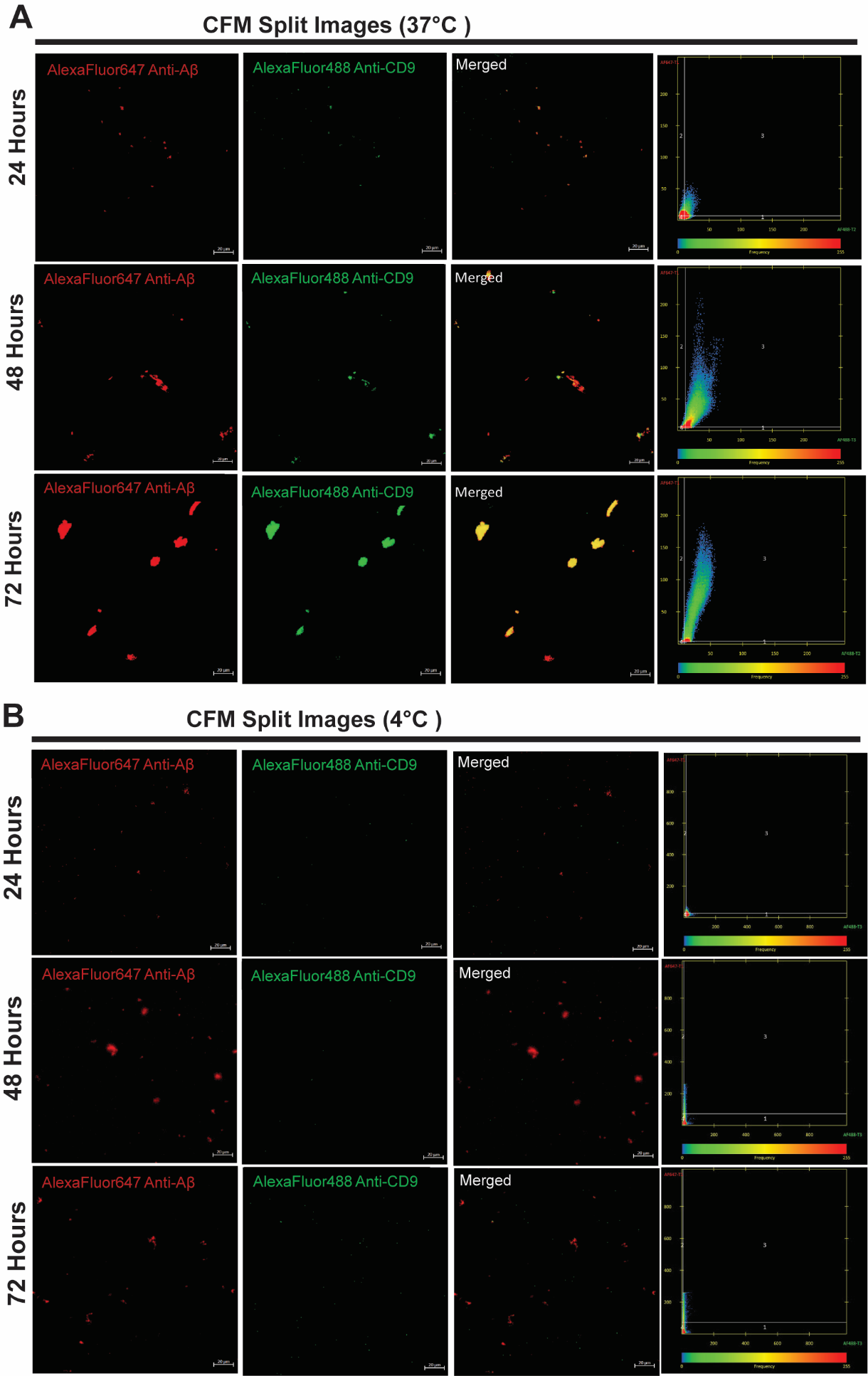


**Supplementary Figure 5:** CFM Split image showing Alexa-Fluor-488 CD9 and Alexa-Fluor-647 Amyloid-β signals of experimental group 24, 48 and 72 hours at 37℃ (A) and at 4℃ (B). Colour yellow is the merged signal of EVs (Green) and Aβ (red). Scaler bar= 20μm.


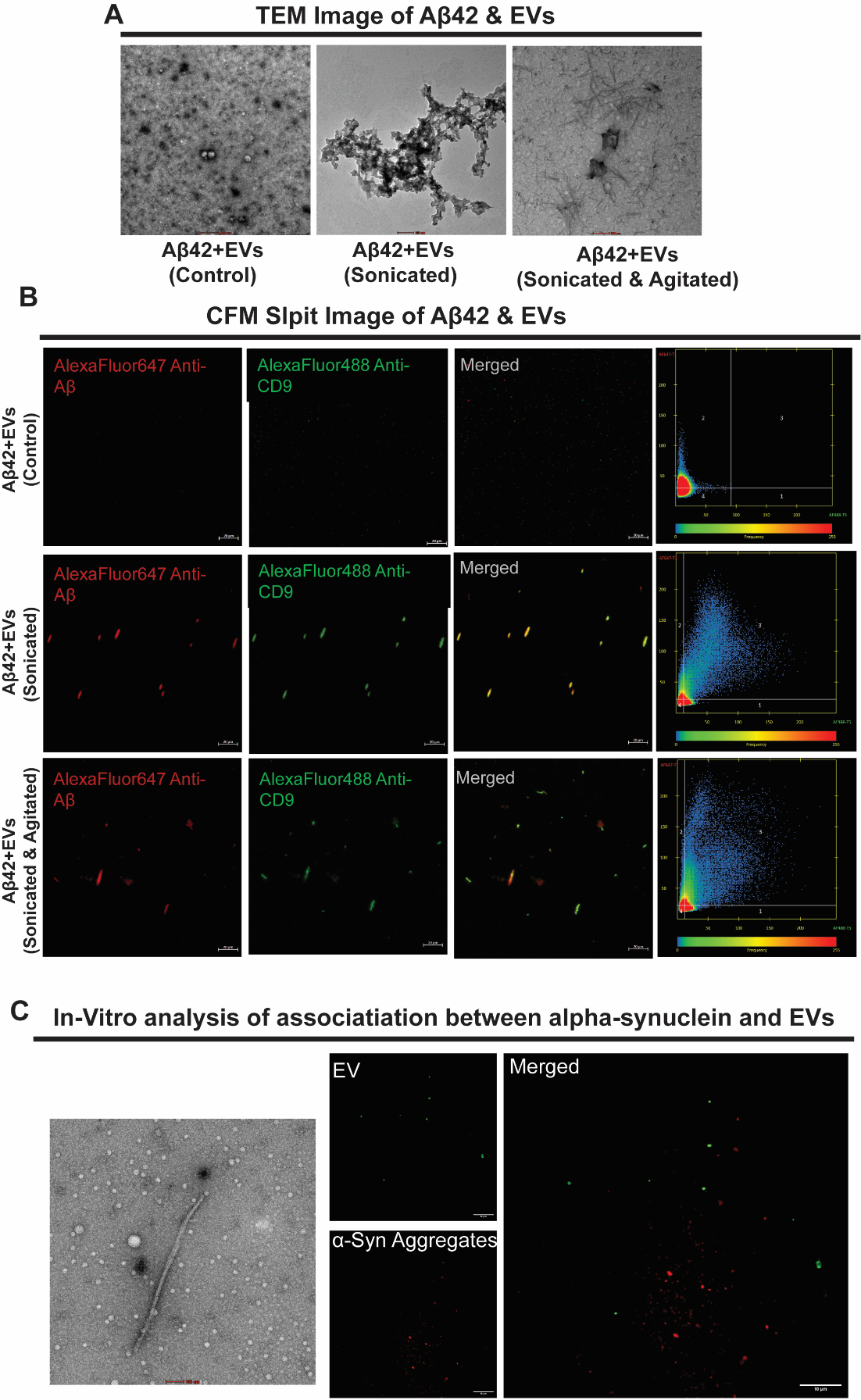


**Supplementary Figure 6:** TEM images of EVs and Aβ in control, sonicated and sonicated & agitated condition (Scale bar 50nm) (A); CFM Split image showing Alexa-Fluor-488 CD9 and Alexa-Fluor-647 Amyloid-β signals for all 3 groups (B). Colour yellow is the merged signal of EVs (Green) and Aβ (red). Scaler bar= 20μm. *In-Vitro* analysis of alpha-synuclein and EVs (C)

**
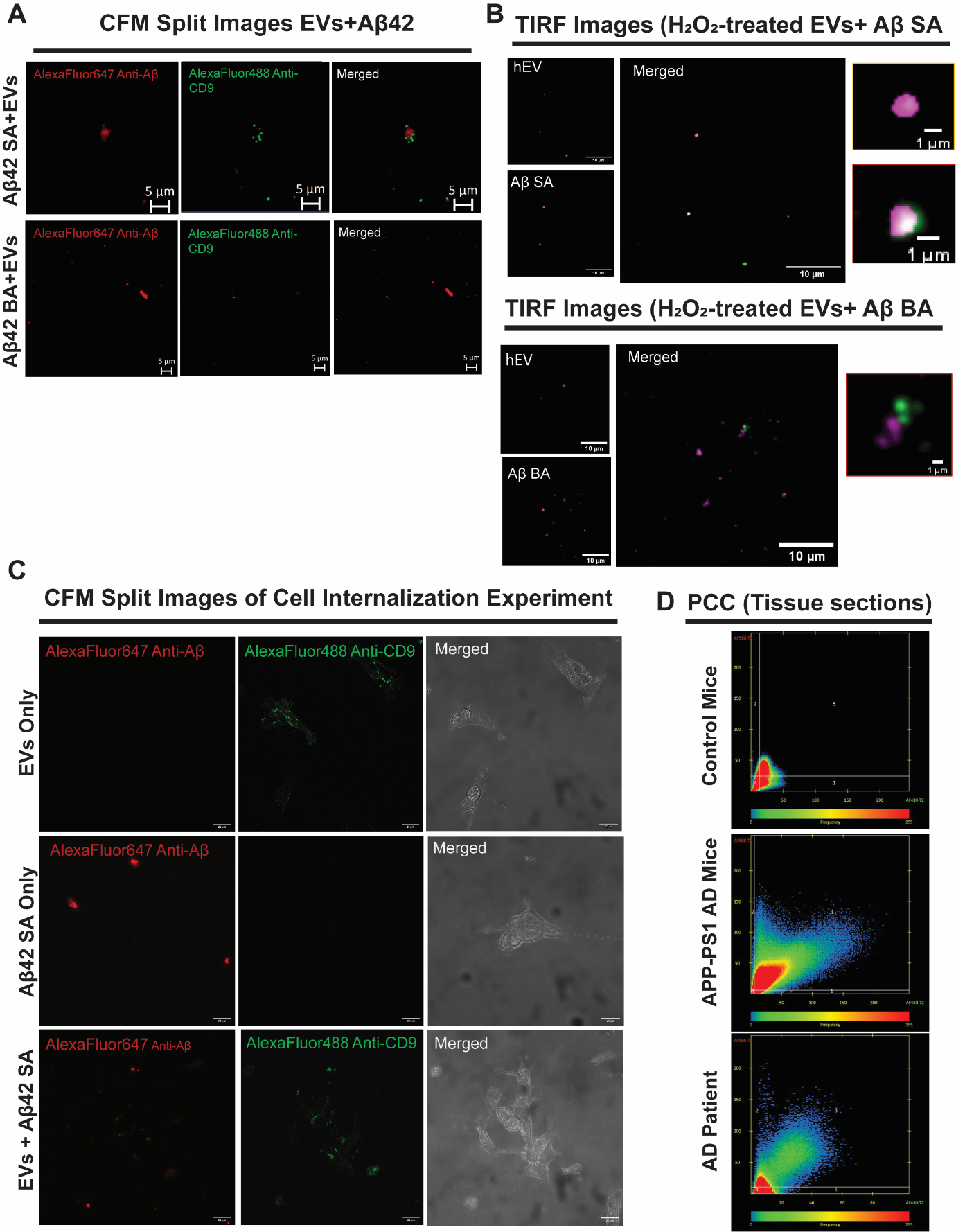
**

**Supplementary Figure 7:** CFM Split image showing Alexa-Fluor-488 CD9 (Green) and Alexa-Fluor-647 Amyloid-β (Red) signals of EVs+ Aβ SA and EVs+ BA under higher magnification shows EVs (Green) sequestering Aβ (Red) (A). TIRF Images of H2O2-treated EVs+ Aβ SA and with BA (B). CFM Split image showing Cell internalisation of Alexa-Fluor-488 CD9 (Green) and Alexa-Fluor-647Amyloid-β (Red) signals of: EVs only; Aβ; and EVs and Aβ together (C). Scale bar= 20μm. PCC Graph (D).


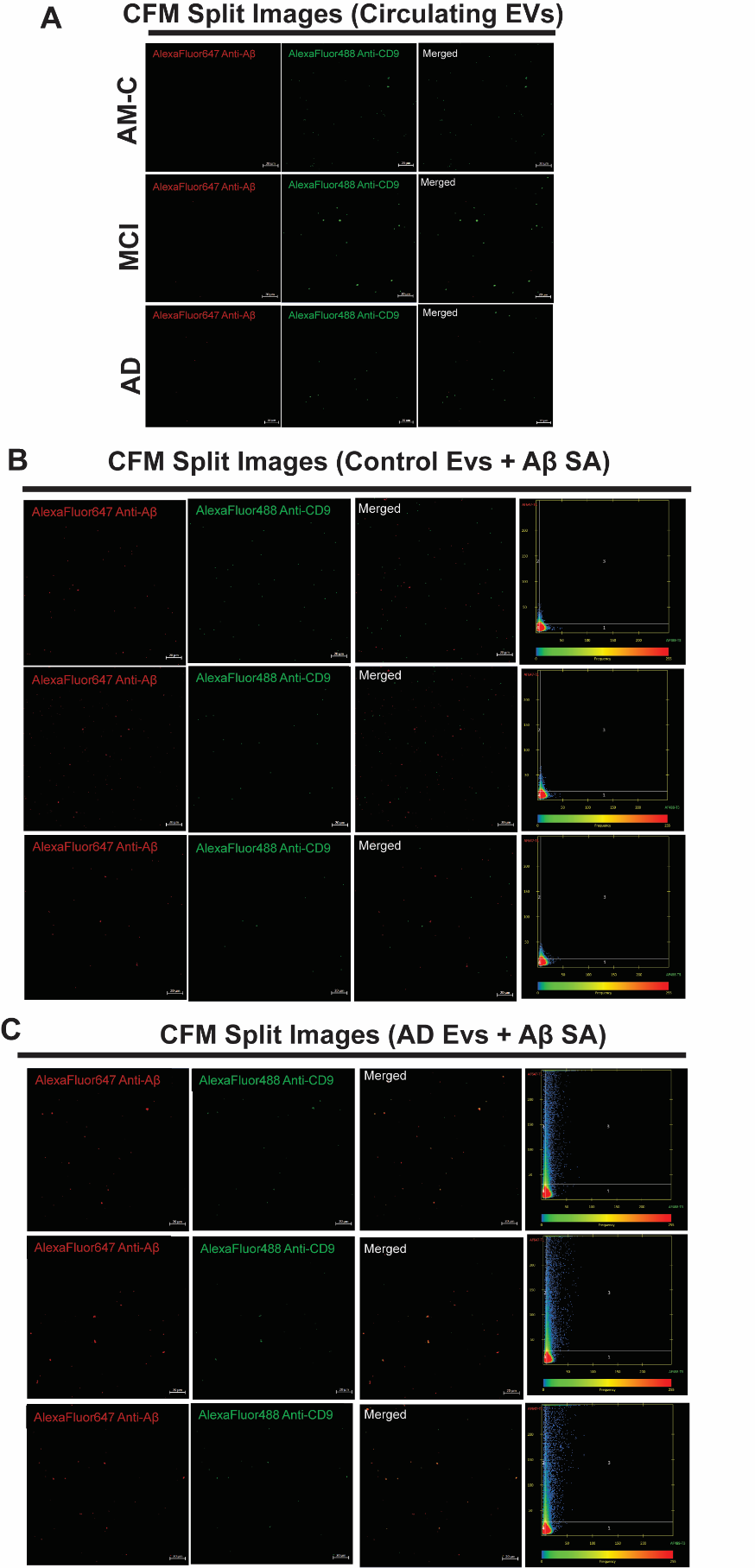


**Supplementary Figure 8:** CFM Split image showing Alexa-Fluor-488 CD9 (Green) and Alexa-Fluor-647Amyloid-β (Red) signals of: Circulating EVs in Age-matched control, MCI and AD patient (A); EVs from healthy control + Aβ SA (B); EVs from AD patient + Aβ SA (C). Scaler bar= 20μm.

|  | **Non-demented Controls**  **(AMC) n=10** | **Mild Cognitive Impairment**  **(MCI) n=3** | **Alzheimer’s Disease**  **(AD) n=9** |
| --- | --- | --- | --- |
| Age (Mean±SEM) | 61.23 ± 1.25 | 73.18 ± 1.05 | 75.01 ± 0.81 |
| Gender Percentage (Male) | 69.7% | 61.08 % | 63.05 % |
| ACE-III ((Mean±SEM)) | ------- | 68.94 ± 2.30 | 39 ± 3.60 |
| MMSE (Mean±SEM) | ------ | 23.92 ± 0.83 | 12.76 ±1.04 |

**Supplementary Table 1:** Demographics Details of the subjects.
