# Supplementary figures and images for: Stress-induced alteration of small extracellular vesicles drives amyloid-beta sequestration and exacerbates Alzheimer’s disease pathogenesis
Source: Alzheimers Res Ther. 2026 Apr 11;18:84. doi: 10.1186/s13195-026-02028-1 (PMC13072553; doi:10.1186/s13195-026-02028-1)

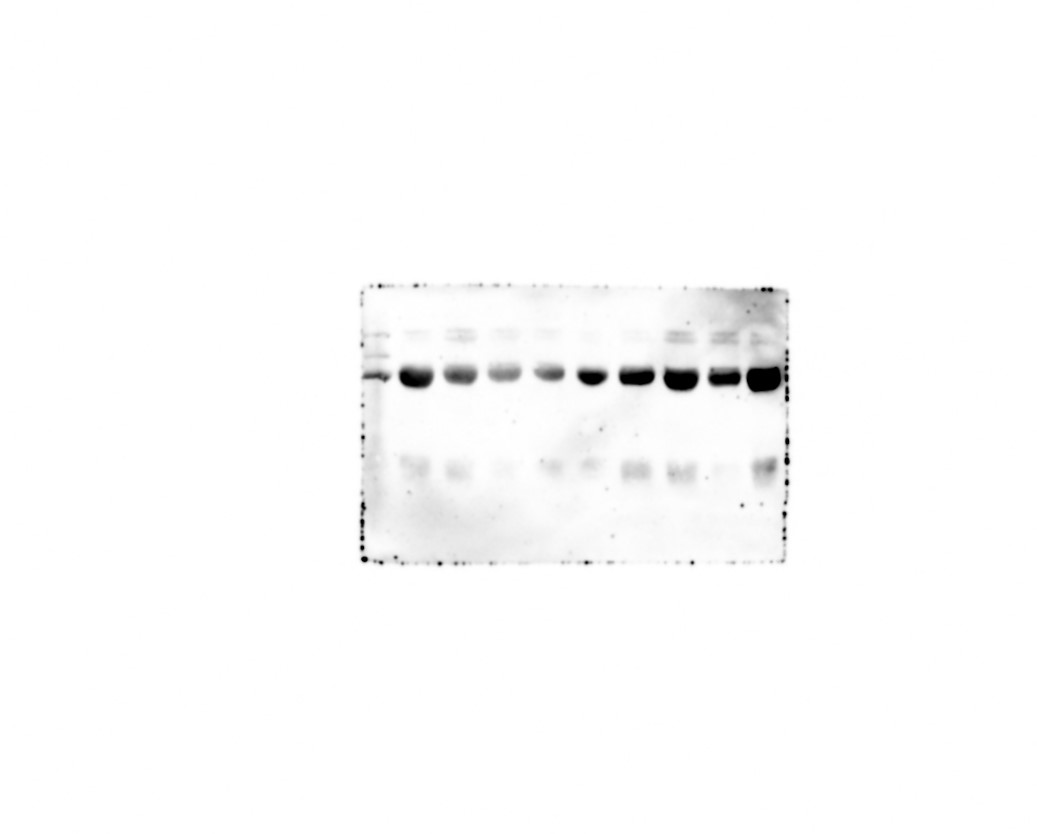

Supplement: Supplementary file 2 — Supplementary Material 2. [file 13195_2026_2028_MOESM2_ESM.jpg]

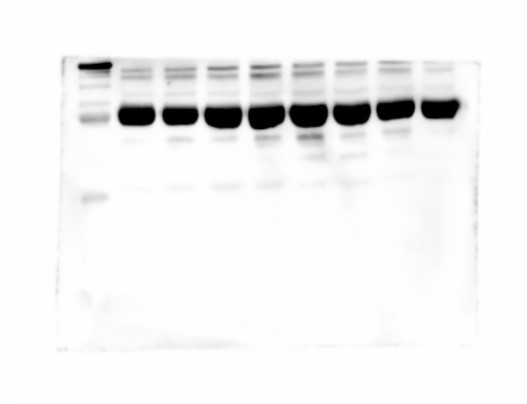

Supplement: Supplementary file 3 — Supplementary Material 3. [file 13195_2026_2028_MOESM3_ESM.jpg]

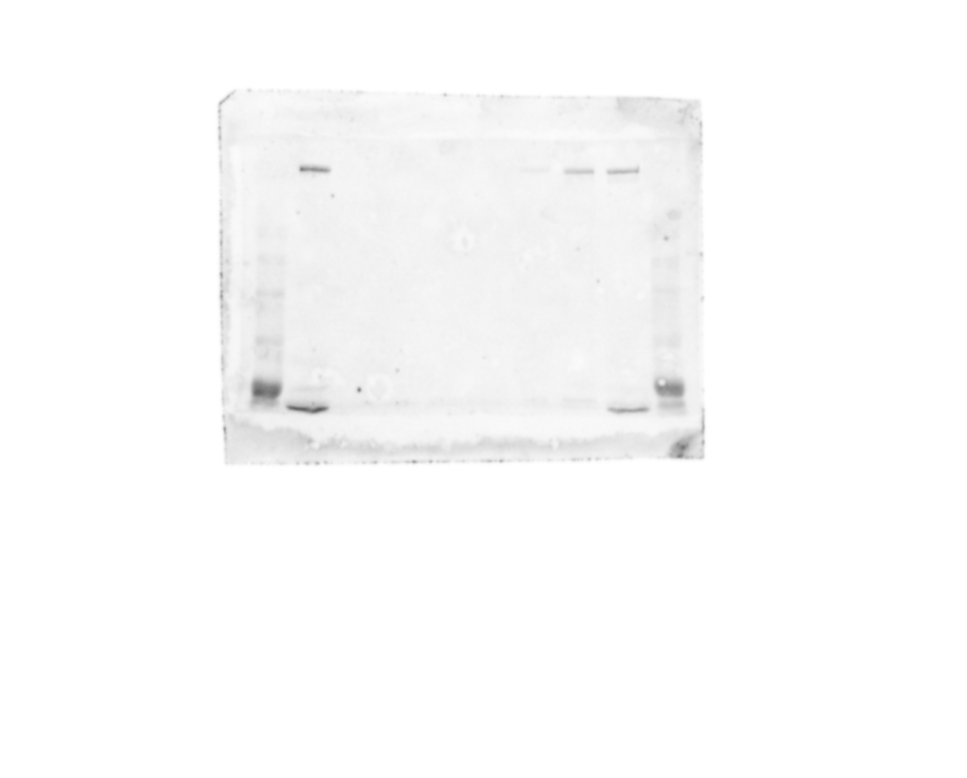

Supplement: Supplementary file 4 — Supplementary Material 4. [file 13195_2026_2028_MOESM4_ESM.tif]

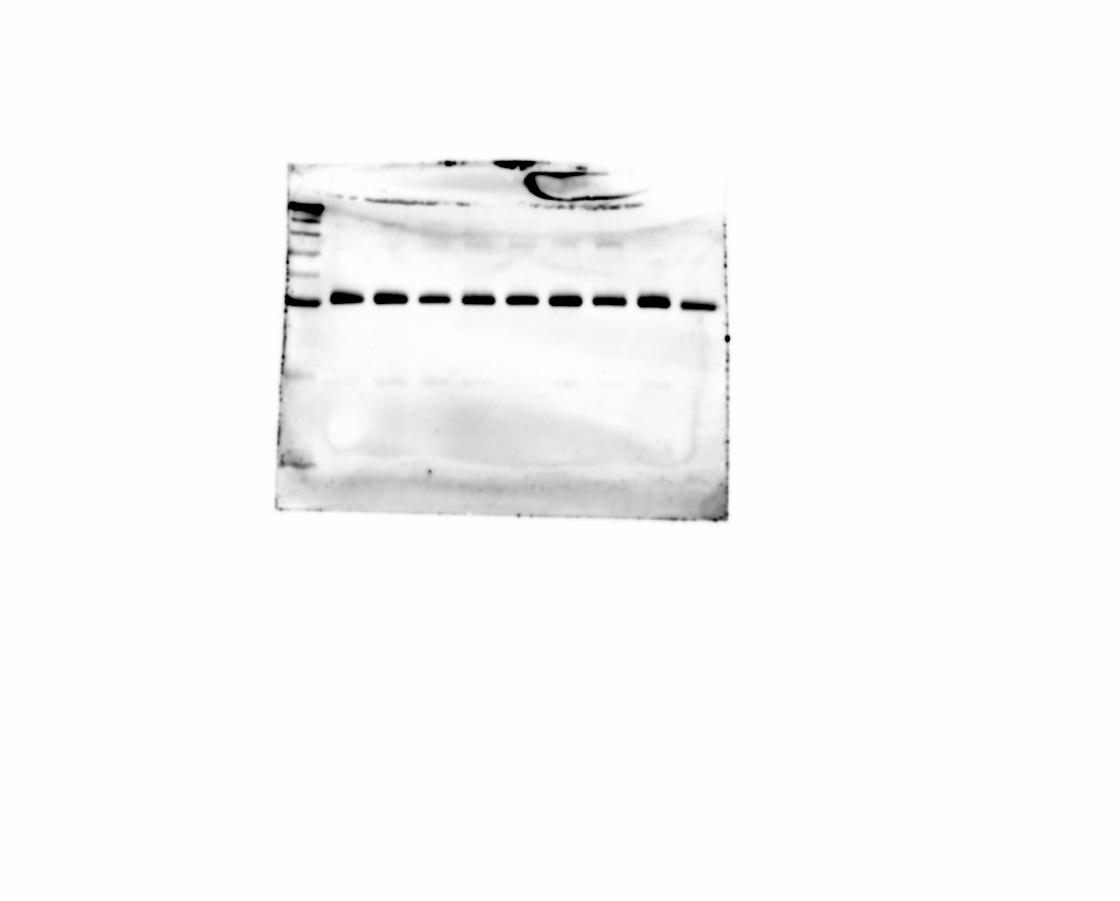

Supplement: Supplementary file 5 — Supplementary Material 5. [file 13195_2026_2028_MOESM5_ESM.jpg]

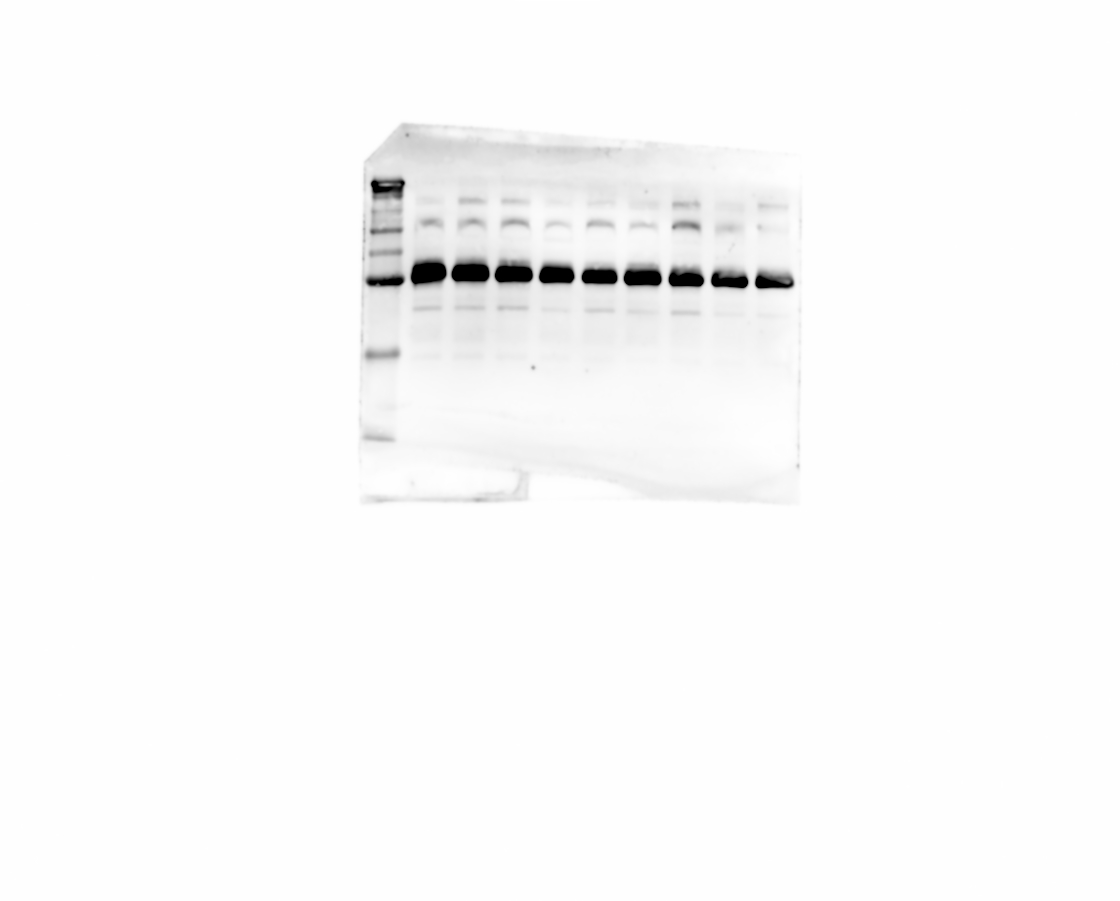

Supplement: Supplementary file 6 — Supplementary Material 6. [file 13195_2026_2028_MOESM6_ESM.jpg]
